# Nuclear receptor Rev‐erbα alleviates intervertebral disc degeneration by recruiting NCoR–HDAC3 co‐repressor and inhibiting NLRP3 inflammasome

**DOI:** 10.1111/cpr.13720

**Published:** 2024-07-24

**Authors:** Qingshuang Zhou, Xiaojiang Pu, Zhuang Qian, Haojie Chen, Nannan Wang, Sinian Wang, Zhenhua Feng, Zezhang Zhu, Bin Wang, Yong Qiu, Xu Sun

**Affiliations:** ^1^ Division of Spine Surgery, Department of Orthopedic Surgery Nanjing Drum Tower Hospital Clinical College of Jiangsu University Nanjing China; ^2^ Division of Spine Surgery, Department of Orthopedic Surgery, Nanjing Drum Tower Hospital, Affiliated Hospital of Medical School Nanjing University Nanjing China

## Abstract

Intervertebral discs (IVDs) are rhythmic tissues that experience daily low‐load recovery. Notably, aging and abnormal mechanical stress predispose IVDs to degeneration due to dysrhythmia‐induced disordered metabolism. Meanwhile, Rev‐erbα acts as a transcriptional repressor in maintaining biorhythms and homeostasis; however, its function in IVD homeostasis and degeneration remains unclear. This study assessed the relationship between low Rev‐erbα expression levels and IVD degeneration. Rev‐erbα deficiency accelerated needle puncture or aging‐induced IVD degeneration, characterized by increased extracellular matrix (ECM) catabolism and nucleus pulposus (NP) cell apoptosis. Mechanistically, Rev‐erbα knockdown in NP cells aggravated rhIL1β‐induced NOD‐like receptor family pyrin domain containing 3 (NLRP3) inflammasome activation, exacerbating the imbalanced ECM and NP cell apoptosis. Meanwhile, blocking NLRP3 inflammasome activation mitigated Rev‐erbα deficiency and needle puncture‐induced IVD degeneration. Particularly, Rev‐erbα mediated the transcriptional repression of the NLRP3 inflammasome via the ligand heme‐binding of nuclear receptor co‐repressor (NCoR) and histone deacetylase 3 (HDAC3) complex. Thus, the increased expression of Rev‐erbα in NP cells following short‐term rhIL1β treatment failed to inhibit NLRP3 transcription in vitro owing to heme depletion. Pharmacological activation of Rev‐erbα in vivo and in vitro alleviated IVD degeneration by altering the NLRP3 inflammasome. Taken together, targeting Rev‐erbα may be a potential therapeutic strategy for alleviating IVD degeneration and its related diseases.

## Graphical abstract

Rev‐erbα deficiency aggravates NP cell apoptosis and imbalance of extracellular matrix. Specifically, pharmacologic Rev‐erbα activation alleviates IVD degeneration via its ligand heme binding to NCoR and HDAC3 corepressor complex to repress NLRP3 inflammasome activation.

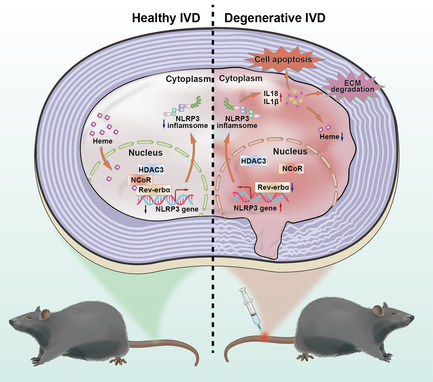

## 1 INTRODUCTION

The human lumbar intervertebral discs (IVDs) are sandwich structures comprising upper and lower endplates, annulus fibrosus, and nucleus pulposus (NP).^1^, ^2^, ^3^, ^4^, ^5^ IVDs are non‐vascular tissues with poor nutrient penetration through the upper and lower endplates to the inner annulus fibrosis and NP tissues.^2^, ^3^, ^4^ NP cells maintain a large extracellular matrix (ECM) volume against a low cell‐to‐ECM ratio.^3^, ^6^ Therefore, with aging and abnormal lifestyle‐induced stress, fragile NP tissues are prone to degeneration, which is associated with low turgor pressure in IVDs. IVD degenerative diseases are increasingly common and are associated with low back pain and poor quality of life.^2^ Hence, understanding the pathology of IVD degeneration is crucial for effectively preventing and treating related diseases.

The IVD is a rhythmic tissue that undergoes a low‐load recovery from daily loading cycles.^1^, ^7^, ^8^ At the molecular level, circadian clock machinery includes and repressorsα.^9^, ^10^, ^11^, ^12^ Transcriptional activators brain and muscle ARNT‐like 1 (BMAL1) and circadian locomotor output cycles kaput (CLOCK) heterodimer induce the expression of transcriptional repressors period and cryptochrome, which subsequently inhibit the activity of BMAL1/CLOCK. BMAL1/CLOCK activates transcription of Rev‐erbs and retinoic acid receptor‐related orphan receptors as the auxiliary loop to maintain the molecular clock. The transcriptional–translational feedback loop system generates circadian oscillations of IVD tissue, temporally maintaining the synchronous physiology of IVDs.^10^, ^12^, ^13^ However, aging and abnormal mechanical stress affect the intrinsic circadian clock of IVDs, which triggers NP cell senescence, cell apoptosis, inflammation, and ECM degradation.^1^, ^7^, ^8^, ^14^ Notably, previous studies on the circadian rhythm of IVDs have focused primarily on the role of BMAL1 in IVD tissues. BMAL1‐deficient mouse models are susceptible to IVD degeneration and low back pain.^1^, ^7^, ^8^, ^14^, ^15^ Meanwhile, the mechanisms underlying the involvement of other vital rhythm genes in the pathogenesis of IVD degeneration remain unclear.

Rev‐erbα is a nuclear receptor 1D subfamily member and acts as a transcriptional repressor. It contributes to the regulation of various cellular processes, including cell differentiation, mitochondrial biogenesis, metabolism, immunity, and inflammation.^9^, ^10^, ^16^, ^17^, ^18^, ^19^ Accordingly, Rev‐erbα deletion in mice is associated with disease pathogenesis.^9^, ^10^, ^12^, ^16^, ^17^, ^18^ For example, Rev‐erbα regulates the circadian activity of the nucleotide binding oligomerization domain‐like receptor family pyrin domain containing 3 (NLRP3) inflammasome to reduce the severity of fulminant hepatitis in mice.^9^ Additionally, Rev‐erbα integrates experimental colitis by repressing the nuclear factor (NF)‐κB/NLRP3 axis.^10^ Notably, targeting Rev‐erbα represents a potential approach for preventing and managing acute inflammation diseases.^16^ Although phagocytosis activates the NLRP3 inflammasome in NP cells to produce inflammatory factors, leading to IVD degeneration, it remains unclear whether Rev‐erbα regulates IVD homeostasis and susceptibility to degeneration via the NLRP3 inflammasome.^1^, ^3^, ^7^ Furthermore, the repressive activity of Rev‐erbα is regulated by binding to heme, promoting recruitment of the nuclear receptor co‐repressor (NCoR) and histone deacetylase 3 (HDAC3) complex.^16^, ^20^, ^21^, ^22^, ^23^ However, whether Rev‐erbα binds to the NCoR–HDAC3 co‐repressor to suppress the NLRP3 inflammasome in NP cells is unclear.

This study identified a crucial role for Rev‐erbα in the pathological progression of IVD degeneration. Rev‐erbα deletion aggravated needle puncture or aging‐induced IVD degeneration by regulating NLRP3 inflammasome activation. Specifically, Rev‐erbα alleviated IVD degeneration via its ligand heme‐binding of the NCoR–HDAC3 complex to repress NLRP3 inflammasome activation. Moreover, pharmacologic Rev‐erbα activation reduced NP cell apoptosis and improved ECM, alleviating IVD degeneration. These findings demonstrate the potential therapeutic value of Rev‐erbα in preventing IVD degeneration.

## 2 EXPERIMENTAL PROCEDURES

### 2.1 Human NP sample collection

Forty‐one fresh human IVD tissue samples were obtained from patients who had undergone spinal surgery at Nanjing Drum Tower Hospital (patient demographic data are provided in Table S1). Samples for detecting tissue proteins were obtained from the first operation in the morning to exclude the effects of rhythmic cycles on expression. The enrolled donors had no autoimmune diseases, endocrine system diseases, or history of hormone use. The Ethics Committee of Nanjing Drum Tower Hospital, Medical School of Nanjing University (No. 2023‐564‐01) approved the project, and the enrolled patients signed informed consent before surgery. All samples were divided into mild IVD degeneration (Pfirrmann Grade I, II, or III) and severe IVD degeneration (Pfirrmann Grade IV or V) groups based on lumbar T2‐magnetic resonance imaging (MRI) images. When NP tissues were isolated during surgery, they were immediately washed with chilled polybutylene succinate (PBS) and frozen with liquid nitrogen; this was conducted for the first 27 patients (Table S1).

### 2.2 Human NP cell culture and transfection

The NP tissues from the remaining patients (Table S1) were sterilized and processed on a clean bench. Tissues were cut into small pieces and digested at 37°C with collagenase II (#C6885‐1G; Sigma, Ohio, USA) for ~2 h. After centrifuging, filtering, and washing, NP cells were dispersed and cultured with 1% penicillin/streptomycin (#15140122, Thermo Fisher Scientific, Massachusetts, USA) and 10% foetal bovine serum (#10099–141, Thermo Fisher Scientific) at 37°C with 5% CO_2_. When the cells achieved ~80% confluency, primary NP cells were digested using trypsin–ethylenediaminetetraacetic acid (EDTA; 0.25%; #25200072, Thermo Fisher Scientific, Massachusetts, USA) and stimulated by 50 ng/mL rhIL‐1β (#SRP6169, Sigma) to simulate an inflammation model of degeneration. Additionally, NP cells were cultured with 10 μM R9009 (#S8692, Selleck, Houston, USA), 10 μM GSK4112 (#SR6452, MCE, Shanghai, China), 5 μM Hemin (#HY‐19424, MCE), and 5 μM MCC950 (#S8930, Selleck).

Lentivirus vectors to interfere with Rev‐erbα expression were constructed by GeneChem (#NM_021724, GeneChem, Shanghai, China), and the cells were transfected according to the manufacturer's instructions. The associated sequences for Rev‐erbα (gene encoding Rev‐erbα) were 5′‐CCAGCCCTGAATCCCTCTATA‐3′ and negative control: 5′‐TTCTCCGAACGTGTCACGT‐3′.

### 2.3 Animals

Wild‐type (WT) C57BL/6 mice were purchased from GemPharmatech (Nanjing, China). All genetically modified mice were generated using a C57BL/6 background. Rev‐erbα^+/−^ heterozygous mice were generated using the CRISPR/Cas9 system (#S‐KO‐05255, Cyagen Biosciences, Inc., Guangzhou, China) (Table S2). The breeding and genotyping strategies have been previously described per the Cyagen user manual. The Rev‐erbα^+/−^ heterozygotes were intercrossed to generate homozygous targeted mice. Age‐ and sex‐matched WT and Rev‐erbα^−/−^ offspring were selected for spontaneous and surgically induced IVD degeneration models. Mice were group‐housed at 20–24°C under a 12‐h dark/12‐h light cycle. All animal care and experimental procedures complied with guidelines approved by the Nanjing First Hospital, Nanjing Medical University (DWSY‐22026152). The mice were randomly allocated to experimental groups based on their body weight.

### 2.4 Coccygeal IVD needle stable models

Twelve‐week‐old mice were used to induce coccygeal IVD needle stable (CINS)‐associated IVD degeneration (Figure S2K). Anaesthesia was induced using isoflurane. To locate the coccygeal IVDs for the needle stab, a body surface location analysis was performed based on intervertebral movement (microscopic observation of the range of motion). Subsequently, minimally invasive incisions were made in the corresponding segments (Co6/7, Co7/8, and Co8/9) of the mouse tail. After clear exposure of the moulding position and adequate haemostasis, a 31‐G needle was vertically inserted into the NP of the corresponding segments through the middle of the IVD (Figure S2J). The needle was advanced 1.5 mm parallel to the endplates. Subsequently, the needle was rotated 180° in the axial direction and held for 10 s. The CINS‐induced model is commonly used to investigate abnormal stress‐induced IVD degeneration.^24^, ^25^ For CINS‐induced degeneration experiments, discs were harvested from Rev‐erbα^−/−^ and WT mice at 6 weeks post‐surgery. In addition, to determine the correlation of IVD degeneration grades with Rev‐erbα expression, mice were subjected to 2, 4, 6, and 8 weeks of stress after needle puncture.

### 2.5 Radiological examinations

The mice were sacrificed by cervical dislocation at 42 days post‐surgery, and spine and coccyx specimens were harvested. The specimens were fixed in 4% paraformaldehyde (#G1101, Servicebio, Wuhan, China) for radiological examination. A micro‐computed tomography (CT) scanner (mCT80; Scanco Medical AG, Switzerland) with a 70 kV as source voltage was used to measure the disc height and vertebral body. The 3D reconstructed image was acquired using Scanco Medical software, and the corresponding virtual x‐ray radiographs were exported. The intervertebral disc height index (DHI) was obtained by dividing the average values of the posterior, middle, and anterior parts of the IVD by the average height of the adjacent vertebral body. Micro‐MRI (Biospec 94/30 USR, Bruker, Germany) was used to evaluate water contents in NP tissues. The relative signal intensity on the T2‐weighted image was calculated using ImageJ software (version 1.8.0_172).

### 2.6 Western blot analyses

Protein lysates were extracted from human NP cells or tissues using radioimmunoprecipitation assay buffer (R0010, Solarbio, Beijing, China) supplemented with 1 mM protease (#329‐98‐6, Solarbio) and 1 mM phosphatase inhibitor (B15002, Bimake, USA). Protein concentrations were determined using a bicinchoninic acid assay Protein Assay Kit (#P0009, Beyotime, Shanghai, China). Protein lysates were loaded, separated via 10% (w/v) sodium dodecyl sulphate‐polyacrylamide gel electrophoresis (#PG112, EpiZyme, Shanghai, China), and transferred onto polyvinylidene fluoride membranes (#IPVH00010, Millipore, USA). After the membranes were blocked with 5% (w/v) milk (#1172GR500, Biofroxx), the membranes were incubated with primary antibodies at 4°C overnight. The primary antibodies are listed in Table S3. An appropriate secondary antibody was used, and signals were detected using a ChemiDocXRS + Imaging System (Tanon, Shanghai, China). Quantitative analysis was performed using ImageJ software.

### 2.7 RNA extraction and quantitative polymerase chain reaction

Total RNA was isolated from human NP cells using RNAiso Plus reagent (Takara Bio) and reverse‐transcribed using an RT reagent kit (Takara Bio). Quantitative polymerase chain reaction (qPCR) was conducted with a total volume of 20 μL using the SYBR Green Q‐PCR Kit (#Q411‐03, Vazyme, Nanjing, China) on a LightCycler 480 PCR System (Roche, Switzerland). The primer sequences are listed in Table S4.

### 2.8 Circadian rhythm of human primary NP cells with Rev‐erbα knockdown

After human primary NP cells were transfected with Rev‐erbα or negative‐control lentivirus vectors, they were incubated in sterile cell culture dishes for 24 h. Next, 100 nM dexamethasone (#HY‐14648; MCE, Shanghai, China) was used to synchronize the cells. After 24 h, RNA was extracted every 4 h seven times (harvested from ZT24 to ZT48). Relative gene expression in different groups was detected using qPCR.

### 2.9 Histological staining

After radiological examinations, specimens were decalcified in 10% EDTA (G1105, Servious, Wuhan, China) for 35 days and subsequently embedded in paraffin. A mid‐coronal‐oriented tissue slice (5 mm) was prepared for safranin O‐fast green (SO&FG) staining using the kit's protocol (G1371, Solarbio).

### 2.10 Immunohistochemistry

Immunohistochemistry (IHC) was performed using standard procedures. Briefly, the sections were de‐paraffinized using xylene and rehydrated with graded ethanol. Enzymatic digestion was performed for antigen retrieval. The slides were blocked with 5% bovine serum albumin (BSA) for 1 h at 37°C and incubated with primary antibodies at 4°C overnight. Sections were washed with PBS and incubated with secondary antibodies for 1 h at room temperature. An ultrasensitive DAB Kit (#1205250, Typing, China) was used for visualization. The primary antibodies are listed in Table S3.

### 2.11 Immunofluorescence

Immunofluorescence (IF) staining of the sections was similar to IHC. Briefly, the tissue sections were de‐paraffinized, rehydrated, antigen‐retrieved, and blocked. Primary antibodies were incubated at 4°C overnight; the sections were immersed in appropriate secondary antibodies and sealed using an antifade mounting medium with 2‐(4‐amidinophenyl)‐6‐indolecarbamidine dihydrochloride (DAPI) (P0131, Beyotime). The NP cells were fixed in 4% paraformaldehyde for 20 min and permeabilized using 0.3% Triton X‐100 (Beyotime) for 20 min. The residual procedures were similar for all tissue sections. Images were captured using a fluorescence microscope (Thunder Imaging Systems, Leica). The primary antibodies are listed in Table S3.

### 2.12 Terminal deoxynucleotidyl transferase dUTP nick end labelling staining

Terminal deoxynucleotidyl transferase dUTP nick end labelling (TUNEL) staining was used to analyse cell apoptosis in IVD tissues with a detection kit (G1501‐100T, Servious) according to the manufacturer's protocol. Briefly, the tissue sections were de‐paraffinized and rehydrated. Next, the sections were incubated with Proteinase K at 37°C for 20 min and equilibration buffer at room temperature for 10 min. Tissues were incubated with a mixed system (recombinant TdTenzyme: FITC‐12‐dUTP labelling mix: equilibration buffer = 1:5:50) at 37°C for 60 min. Finally, the sections were sealed using an antifade mounting medium containing DAPI (P0131, Beyotime) and visualized with a fluorescence microscope (Thunder Imaging Systems, Leica).

### 2.13 Flow cytometry

Flow cytometry (FCM) was used to analyse the apoptosis of NP cells after different treatments with an Annexin V‐Alexa Fluor 647/PI Apoptosis Assay Kit (FMSAV647‐100, FcMACS, NanJing, China). Briefly, cells were digested with trypsin without EDTA. After centrifugation, the cells were resuspended in 100 μL of binding buffer and incubated with 10 μL of propidium iodide (PI) and 5 μL of rh Annexin V‐Alexa Fluor 647 at room temperature for 15 min away from light. Finally, 400 μL of PBS was added, and the samples were analysed using a BD AccuriC6 Plus Flow CytoMeter (BD Biosciences, USA). Data were analysed using FlowJo software (version 10.8.1).

### 2.14 Co‐immunoprecipitation

Human primary NP cells were cultured in sterile cell culture dishes for 24 h and synchronized with dexamethasone. They were then divided into the control, rhIL‐1β, and rhIL‐1β + hemin groups. After culturing for 48 h, whole lysates from the three groups were used for co‐immunoprecipitation (CoIP) analyses. Cells were collected in lysis buffer (50 mM Tris [pH 7.5], 150 mM NaCl, 2 mM EDTA, 1.5% NP40, 1× Complete [Roche], and 1 mM phenylmethanesulfonyl fluoride). Next, 1 μg of Rev‐erbα or control IgG antibodies were incubated with lysate at 4°C overnight. Protein G Agarose Beads (CST, Danvers, USA, 37478) were used to bind the antigen–protein complex. The immunoprecipitated proteins were analysed using western blotting.

### 2.15 Statistical analyses

Statistical analyses were conducted using the GraphPad Prism software (version 9.0.0.121). Orthographic distribution data were expressed as means ± standard deviation (SD). Student's *t*‐test was used to compare two groups, and a one‐way analysis of variance (ANOVA) followed by Fisher's least significant difference post hoc test was performed to assess the statistical significance among more than two groups. Multivariate ANOVA was used to assess the statistical significance of more than one variable. Statistical significance was set at *p* < 0.05.

## 3 RESULTS

### 3.1 Downregulation of Rev‐erbα in human and mouse degenerative NP tissues

**FIGURE 1:**
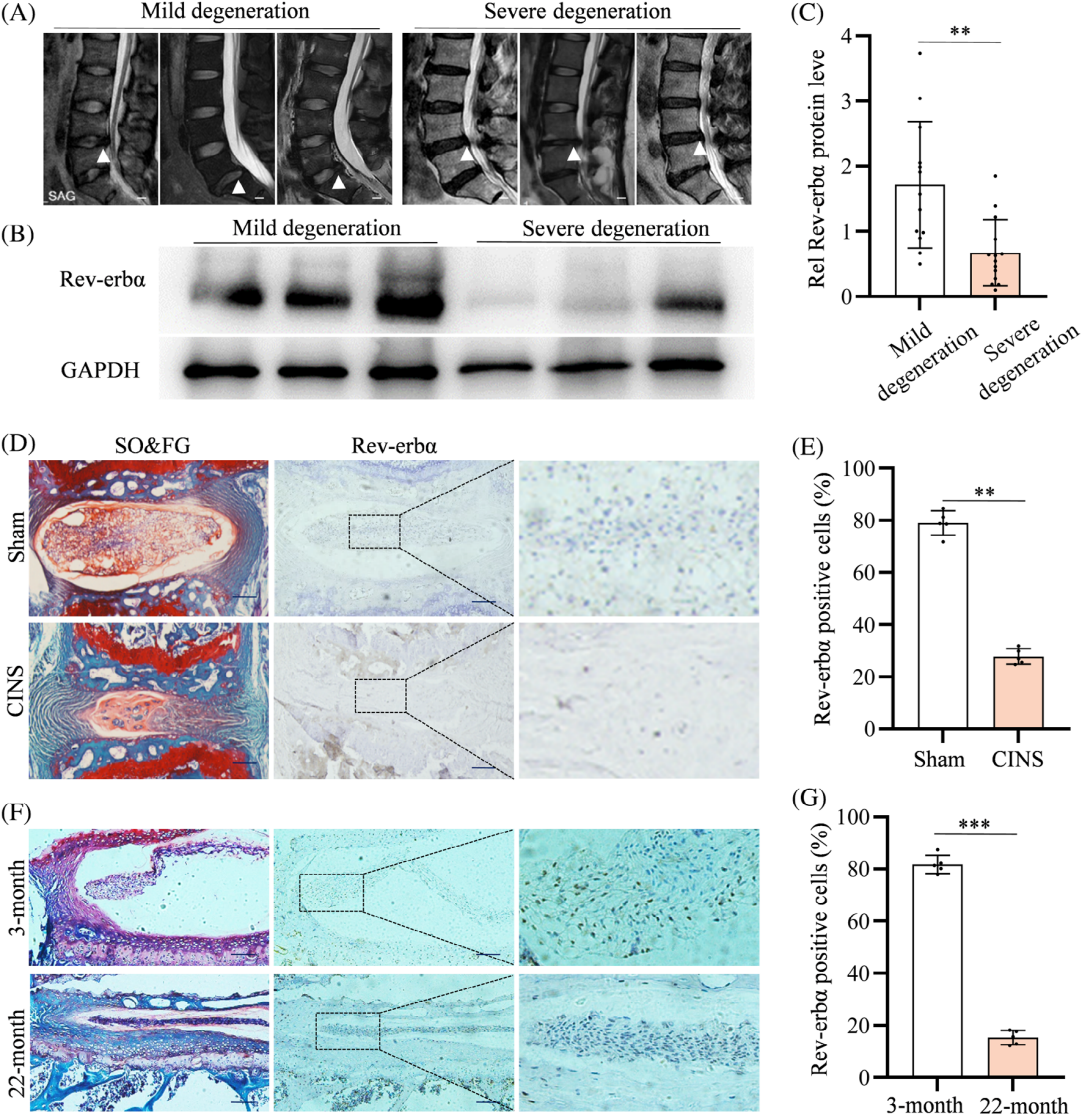
Downregulation of Rev‐erbα in human nucleus pulposus (NP) tissues of severe IVD degeneration and NP tissues of aging mice or needle puncture induced mice. (A) Representative magnetic resonance imaging (MRI) images of mild IVD degeneration (Pfirrmann Grade I/II/III) and severe IVD degeneration (Pfirrmann grade IV/V). White triangles: Operative level. Scale bar: 1 cm (B,C) Western blot analyses of Rev‐erbα in human NP tissues of mild and severe IVD degeneration; *n* = 13 (mild IVD degeneration) and 14 (severe IVD degeneration). (D,E) Safranin O & Fast Green (SO&FG) and immunohistochemistry (IHC) staining of Rev‐erbα for sham and coccygeal IVD needle stable (CINS)‐induced IVD degeneration for 6 weeks; *n* = 5. Scale bar: 100 μm. (F,G) SO&FG and IHC staining of Rev‐erbα for young (3‐month‐old) and aged (22‐month‐old) mouse IVDs; *n* = 5. Scale bar: 100 μm. IVD, intervertebral disc. Student's *t*‐test was used to assess statistical significance. *p < 0.05, **p < 0.01, ***p < 0.001.

To explore the potential role of Rev‐erbα in the pathogenesis of IVD degeneration, we assessed its expression in human NP tissues, which play a vital role in dispersive stress exerted on IVDs. Western blot (WB) analyses revealed that the abundance of Rev‐erbα in NP tissues was statistically reduced in the severe IVD degeneration (Pfirrmann Grade IV/V) group compared with that in the mild IVD degeneration (Pfirrmann Grade I/II/III) group (Figure 1A–C).

The CINS‐induced mouse model was further employed to evaluate the expression change of Rev‐erbα after IVD degeneration. SO&FG staining of IVD sections revealed decreased amounts of proteoglycans (PGs) and increased fibrosis in the NP tissues of mice that underwent CINS for 6 weeks compared with those in the sham group (Figure 1D). IHC staining showed a decrease in Rev‐erbα abundance within the NP tissues of mice that underwent CINS (Figure 1D,E); this was confirmed by IF staining (Figure S1A,B). Furthermore, to identify the changes in Rev‐erbα expression within NP tissues induced by CINS at different times, IVDs of mice were harvested after CINS for 2, 4, 6, and 8 weeks, respectively. The Rev‐erbα expression was lower in NP tissues after CINS for 6 weeks than in those after CINS for 2 or 4 weeks and similar to that for 8 weeks (Figure S1E,F).

The expression of Rev‐erbα in the IVDs of 3‐month‐old mice and 22‐month‐old mice was detected to assess changes in IVDs with aging. SO&FG and IHC staining indicated decreased PG and Rev‐erbα abundance, respectively, in the NP tissues of aged (22‐month‐old) mice compared with that of young (3‐month‐old) mice (Figure 1F,G). IF staining confirmed these changes in Rev‐erbα abundance within IVDs with aging (Figure S1C,D). These data confirm that Rev‐erbα is downregulated in human and mouse degenerative NP tissues.

### 3.2 Rev‐erbα deletion results in progressive IVD degeneration in needle puncture‐induced mice

Rev‐erbα knockout (Rev‐erbα^−/−^) mouse models were established via CRISPR/Cas9 technology to investigate its effect on IVDs. The 4‐month‐old Rev‐erbα^−/−^ mice were smaller than WT mice, whose genotype was confirmed via PCR (Figure S2A,B). Additionally, IF staining indicated significantly decreased Rev‐erbα levels in the coccygeal IVDs of Rev‐erbα^−/−^ mice (Figure S2C,D). Nevertheless, the IVD degenerative markers between Rev‐erbα^−/−^ and WT IVDs without operation did not differ significantly (Figure S2E–J). Specifically, micro‐CT revealed a similar DHI percentage between Rev‐erbα^−/−^ and WT IVDs (Figure S2E,F). SO&FG staining revealed comparable PG abundance (Figure S2G,H), while IF staining exhibited equal aggrecan (ACAN) and matrix metalloproteinase 13 (MMP13) levels between the two groups (Figure S2I,J).

**FIGURE 2:**
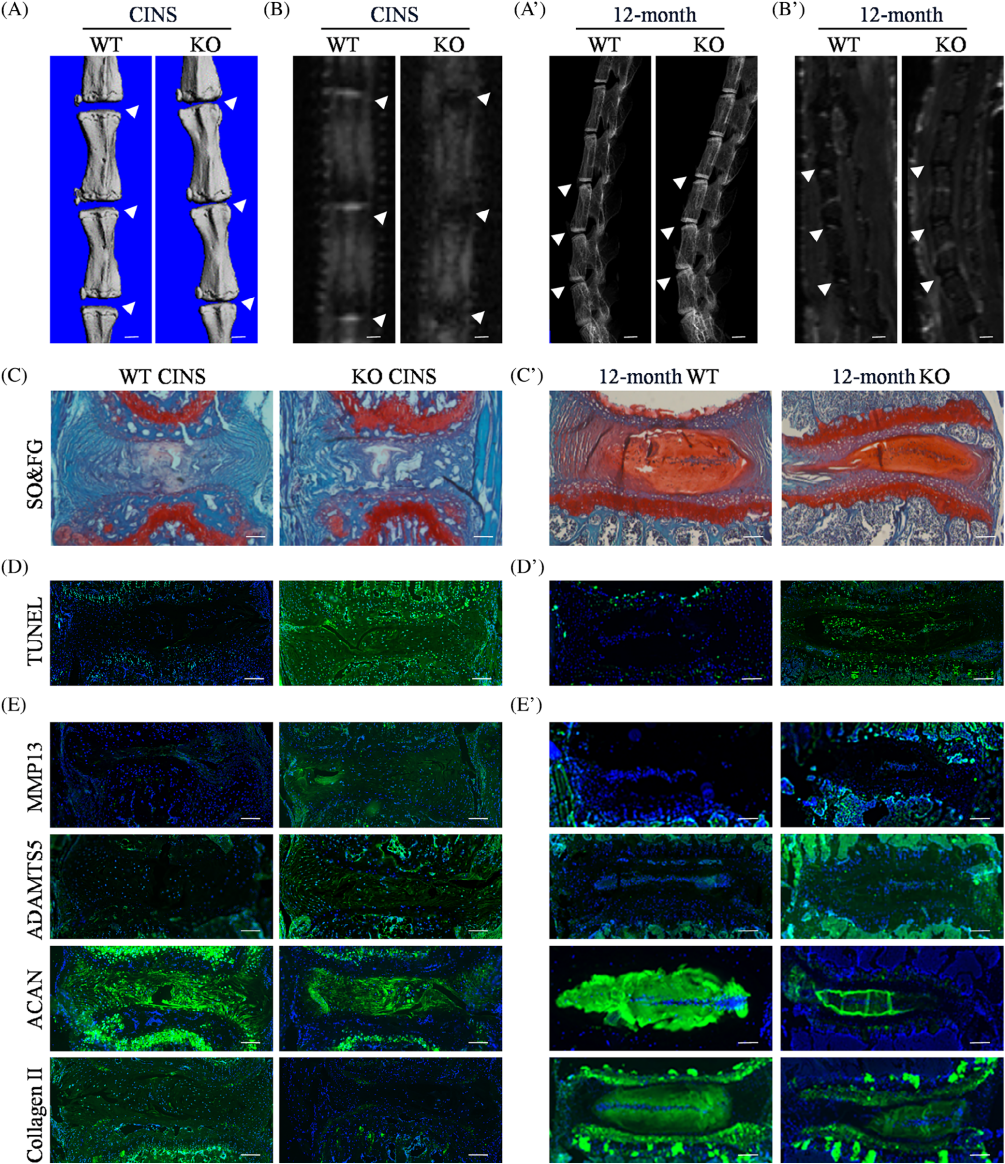
Rev‐erbα deletion accelerates progressive degeneration in needle puncture induced coccygeal intervertebral discs (IVDs) and aged lumbar IVDs. (A) Representative micro‐computed tomography (CT) scan of coccygeal IVD needle stable (CINS)‐induced coccygeal vertebrae of WT and Rev‐erbα^−/−^ mice. White triangles: Coccygeal IVDs; *n* = 5. Scale bar: 1 mm. (B) Representative micro‐magnetic resonance imaging (MRI) scan of CINS‐induced coccygeal vertebrae of WT and Rev‐erbα^−/−^ mice. White triangles: Coccygeal IVDs; *n* = 5. Scale bar: 1 mm. (C) Safranin O & Fast Green (SO&FG) staining for CINS‐induced coccygeal vertebrae of wild type (WT) and Rev‐erbα^−/−^ mice; *n* = 5. Scale bar: 100 μm. (D) Terminal deoxynucleotidyl transferase dUTP nick end labelling (TUNEL) staining for CINS‐induced coccygeal vertebrae of WT and Rev‐erbα^−/−^ mice; *n* = 5. Scale bar: 100 μm. (E) Immunofluorescence (IF) staining for MMP13, ADAMTS5, ACAN, and collagen II in CINS‐induced coccygeal vertebrae of WT and Rev‐erbα^−/−^ mice; *n* = 5. Scale bar: 100 μm. (A') Representative micro‐CT scans of lumbar IVDs (L3/4, L4/5, L5/6) from 12‐month‐old WT and Rev‐erbα^−/−^ mice. White triangles: Lumbar IVDs; *n* = 5. Scale bar: 2 mm. (B') Representative micro‐MRI scan of lumbar IVDs (L3/4, L4/5, L5/6) from 12‐month‐old WT and Rev‐erbα^−/−^ mice. White triangles: Lumbar IVDs; *n* = 5. Scale bar: 2 mm. (C') SO&FG staining of lumbar IVDs from 12‐month‐old WT and Rev‐erbα^−/−^ mice; *n* = 5. Scale bar: 100 μm. (D') TUNEL staining of NP tissues from 12‐month‐old WT and Rev‐erbα^−/−^ mice; *n* = 5. Scale bar: 100 μm. (E') IF staining of MMP13, ADAMTS5, ACAN, and collagen II from 12‐month‐old WT and Rev‐erbα^−/−^ mice; *n* = 5. Scale bar: 100 μm.

In this study, the coccygeal IVDs of 3‐month‐old mice were subjected to CINS or sham operation for 6 weeks to examine the effect of Rev‐erbα on IVD degeneration. Micro‐CT revealed a reduced DHI percentage in CINS‐induced IVDs; moreover, this effect was significantly aggravated by Rev‐erbα deficiency (Figures S2E,F, 3A, and 2A). Micro‐MRI revealed that water contents of NP tissues were decreased in CINS induced Rev‐erbα^−/−^ mice compared with those in WT mice (Figures 2B and S3B). Based on the morphological SO&FG staining and histological scores, CINS induced severe IVD destruction with lower PG levels; these effects were amplified by Rev‐erbα deficiency (Figures S2G,H, S3C, and 2C). Quantitative analyses of TUNEL staining showed a higher incidence of cell apoptosis in the CINS‐induced IVD degeneration of Rev‐erbα^−/−^ mice (Figures 2D and S3D). IF staining revealed decreased levels of anabolic ECM components, ACAN and collagen II, and increased levels of catabolic ECM components, MMP13 and disintegrin and metalloproteinase with thrombospondin motif 5 (ADMTS5), in Rev‐erbα^−/−^ IVDs (Figures S2D,G, S3E, and 2E). Collectively, these data indicate that Rev‐erbα deletion accelerates the progression of coccygeal IVD degeneration induced by needle puncture.

### 3.3 Rev‐erbα deletion accelerates spontaneous lumbar IVD degeneration in aged mice

In addition to needle puncture, aging is associated with the progression of IVD degeneration.^1^, ^3^, ^4^, ^7^, ^24^, ^26^ We used 12‐month‐old mice with or without Rev‐erbα^−/−^ and investigated long‐term IVD homeostasis. Based on the morphological and histological changes between groups, micro‐CT revealed a significantly reduced DHI percentage in the lower lumbar (L3–6) IVDs of Rev‐erbα^−/−^ mice (Figures 2A' and S3A'); micro‐MRI revealed lower water contents in the NP tissues of lower lumbar IVDs in Rev‐erbα^−/−^ mice (Figures 2B' and S3B'). SO&FG staining indicated a moderate effect on 12‐month‐old mutant discs, as evidenced by few NP cells and the loss of demarcation between the NP and annulus fibrosis compartments (Figures 2C' and S3C'). TUNEL staining further revealed deteriorative cell apoptosis of 12‐month‐old mutant discs (Figures 2D' and S3D'). Quantitative IF staining analyses showed that 12‐month‐old Rev‐erbα^−/−^ mice were characterized by decreased ACAN and collagen II and increased MMP13 and ADAMTS5 levels (Figures 2E' and S3E'). Notably, 12‐month‐old Rev‐erbα^−/−^ mice exhibited an aging phenotype with slow movements and back bulge (Videos S1a and S1b). As revealed using micro‐CT and overfitting x‐rays, 12‐month‐old Rev‐erbα^−/−^ mice were characterized by a larger thoracolumbar kyphosis (Figure S4A). Micro‐MRI of the whole spine revealed severe IVD degeneration and decreased mass of paravertebral muscles of 12‐month‐old Rev‐erbα^−/−^ mice (Figure S4B). We evaluated changes in the human degenerative spine based on full spinal images (Figure S4C) and found that Rev‐erbα^−/−^ mice exhibited aging and accelerated progression of lumbar IVD degeneration.

### 3.4 Rev‐erbα deletion promotes ECM catabolism and cell apoptosis via NLRP3 inflammasome in primary human NP cells

Next, we investigated whether Rev‐erbα regulates the NLRP3 inflammasome in NP cells. Notably, NLRP3 oscillated opposite to Rev‐erbα, indicating that NLRP3 is a potential clock‐controlled gene in human primary NP cells (Figure S5A). Rev‐erbα knockdown by lentivirus (shRev‐erbα) promoted higher expression of NLRP3 and restrained its rhythmicity. The NLRP3 expression pattern was similar to that of BMAL1, which Rev‐erbα directly restricts. Furthermore, quantitative IF staining revealed significantly increased NLRP3 expression in 12‐month‐old Rev‐erbα^−/−^ mouse NP tissues compared with that in WT mouse NP tissues (Figure S5B,C). We speculate that NLRP3 is a clock‐controlled gene and a direct target of Rev‐erbα in human NP cells.

**FIGURE 3:**
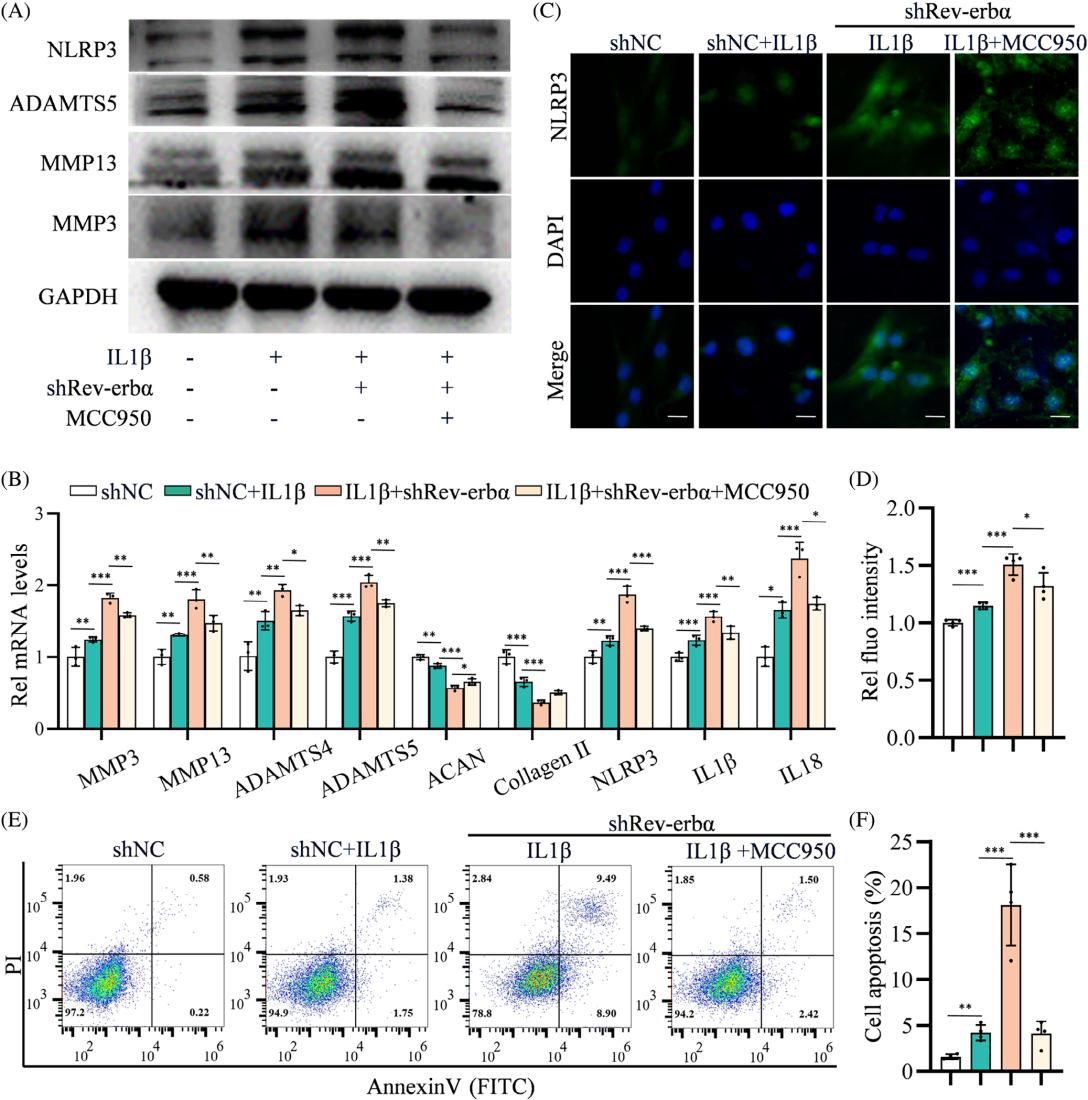
Rev‐erbα deletion promotes extracellular matrix (ECM) catabolism and cell apoptosis by promoting NLRP3 inflammasome activation in human primary nucleus pulposus (NP) cells. (A) Western blotting analyses of NLRP3, MMP3, MMP13, and ADAMTS5 in NP cells transfected with negative control (shNC) or Rev‐erbα knockdown lentivirus (shRev‐erbα), pre‐treated with or without MCC950 and treated with or without 50 ng/mL rhIL‐1β for 44 h; *n* = 3. (B) Quantitative polymerase chain reaction (qPCR) analyses of MMP3, MMP13, ADAMTS4, ADAMTS5, ACAN, collagen II, NLRP3, IL1β, and IL18 in NP cells as treated in (A); *n* = 3. (C,D) Immunofluorescence (IF) staining for NLRP3 localization and intensity levels in NP cells as treated in (A); *n* = 4. Scale bar: 20 μm. (E,F) Flow cytometry analyses of apoptosis rate of NP cells as treated in (A); *n* = 4. Student's *t*‐test or multivariate analysis of variance (ANOVA) was performed to assess statistical significance. NS, no statistical significance. *p < 0.05, **p < 0.01, ***p < 0.001.

In vitro, NLRP3 expression was increased in NP cells with the cultivation of rhIL1β, which was further increased by shRev‐erbα (Figure 3A). Furthermore, NP cell culture with rhIL1β triggered off increased catabolism of MMP3, MMP13, and ADAMTS5, and shRev‐erbα further aggravated the catabolism markers (Figure 3A). qPCR revealed increased levels of catabolism markers (MMP3, MMP13, ADAMTS4, ADAMTS5) and inflammation markers (NLRP3, IL1β, IL18) and decreased levels of metabolism markers (collagen II, ACAN) in NP cells treated with rhIL1β, which was aggravated in NP cells induced by shRev‐erbα and rhIL1β (Figure 3B). IF staining showed deterioration of NLRP3 expression in shRev‐erbα‐treated NP cells compared with that in those cultured solely with rhIL1β (Figure 3C,D). FCM analyses demonstrated that shRev‐erbα aggravated NP cell apoptosis under rhIL1β culture conditions (Figure 3E,F). The NP cells' degenerative phenotypes induced by shRev‐erbα and rhIL1β, including ECM catabolism, inflammation, and cell apoptosis, could be partially reversed by MCC950—a specific inhibitor of NLRP3 inflammasome (Figure 3A–F). These data indicate that Rev‐erbα knockdown aggravates the rhIL1β‐induced IVD degenerative phenotype in vitro by promoting NLRP3 inflammasome activation.

### 3.5 NLRP3 inflammasome inhibitors limit the progression of IVD degeneration caused by needle puncture in Rev‐erbα deletion mice

**FIGURE 4:**
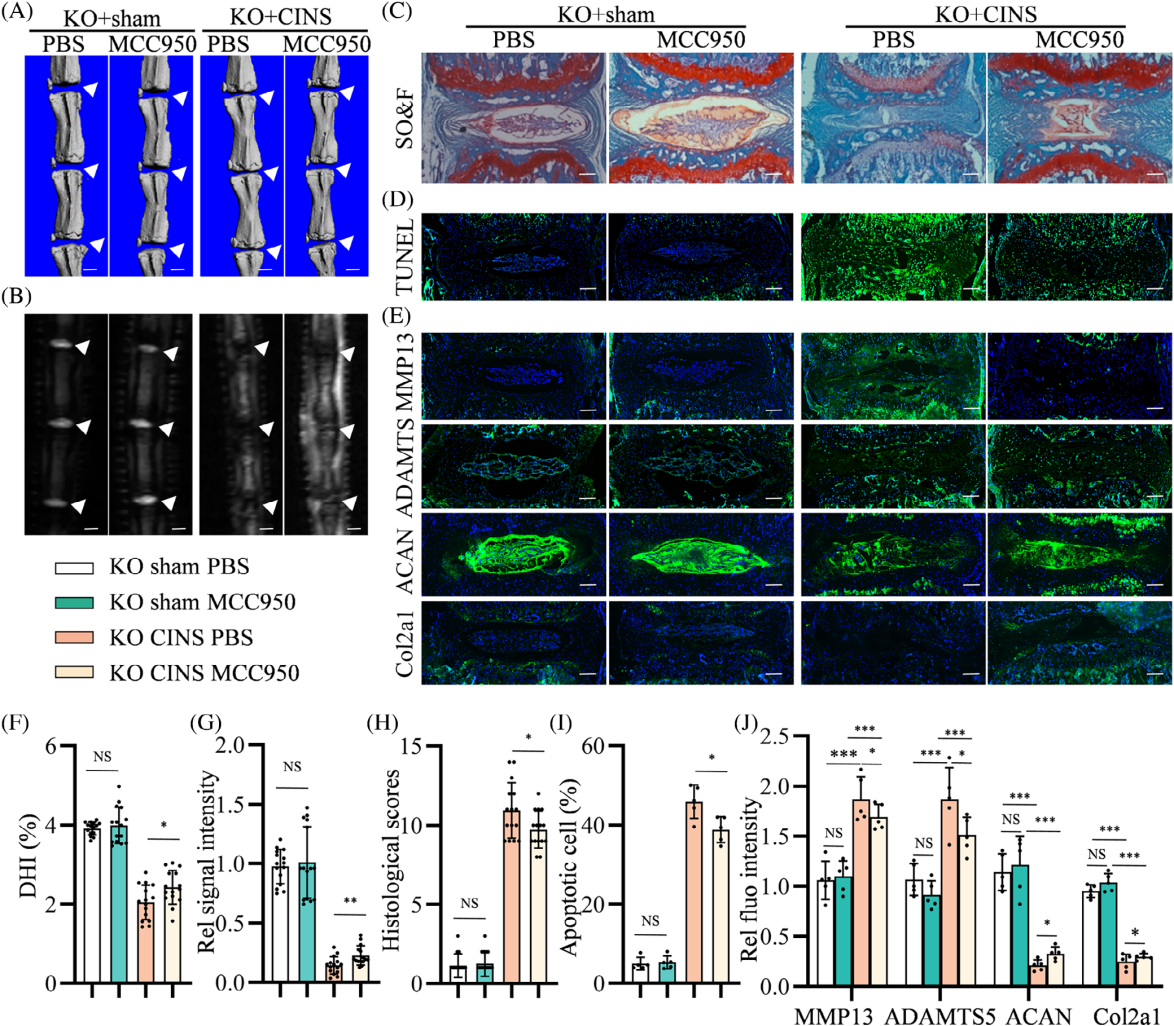
NLRP3 inflammasome inhibitor limits the progression of intervertebral disc (IVD) degeneration caused by needle puncture in Rev‐erbα^−/−^ mice. (A,F) Representative micro‐computed tomography (CT) scans and DHI% of Rev‐erbα^−/−^ mouse coccygeal IVDs with or without coccygeal IVD needle stable (CINS)‐induced IVD degeneration and treated with MCC950 or polybutylene succinate (PBS); *n* = 5. White triangles: Coccygeal IVDs. Scale bar: 1 mm. (B,G) Representative micro‐magnetic resonance imaging (MRI) scan and quantification of the relative signal intensity of Rev‐erbα^−/−^ mouse IVDs as treated in (A); *n* = 5. White triangles: Coccygeal IVDs. Scale bar: 1 mm. (C,H) Safranin O & Fast Green (SO&FG) staining and histological scores of Rev‐erbα^−/−^ mouse IVDs as treated in (A); *n* = 5. Scale bar: 100 μm. (D,I) Apoptosis detected using terminal deoxynucleotidyl transferase dUTP nick end labelling (TUNEL) staining of Rev‐erbα^−/−^ mouse IVDs as treated in (A); *n* = 5. Scale bar: 100 μm. (E,J) Expression of MMP13, ADAMTS5, ACAN, and collagen II detected using immunofluorescence of Rev‐erbα^−/−^ mouse IVDs as treated in (A). Multivariate analysis of variance was used to assess statistical significance. NS, no statistical significance. *p < 0.05, **p < 0.01, ***p < 0.001.

To further assess whether Rev‐erbα deficiency exacerbated IVD degeneration in vivo via NLRP3 inflammasome activation, 3‐month‐old Rev‐erbα^−/−^ mice were subjected to CINS or sham surgery. These mice were further grouped according to the intraperitoneal administration of MCC950 or PBS for 6 weeks. As revealed by micro‐CT and micro‐MRI, injection of MCC950 improved the DHI and water contents in the CINS‐induced IVDs of Rev‐erbα^−/−^ mice compared with PBS injection (Figure 4A,B,F,G). SO&FG staining revealed that MCC950 reduced PG loss in CINS‐treated IVDs (Figure 4C,H). TUNEL staining further revealed that MCC950 reduced NP cell apoptosis (Figure 4D,I) and improved the ECM metabolism and catabolism markers (Figure 4E,J) of Rev‐erbα^−/−^ NP tissues in CINS‐induced IVD degeneration. Comparative experiments did not detect significant changes in the mice administered MCC950 or PBS that underwent sham surgery (Figure 4A–J). These data demonstrate that CINS‐induced IVD degeneration can be resisted in Rev‐erbα‐deficient mice by inhibiting the NLRP3 inflammasome.

### 3.6 Rev‐erbα recruits the NCoR and HDAC3 co‐repressor by hemin to inhibit the transcription of NLRP3

**FIGURE 5:**
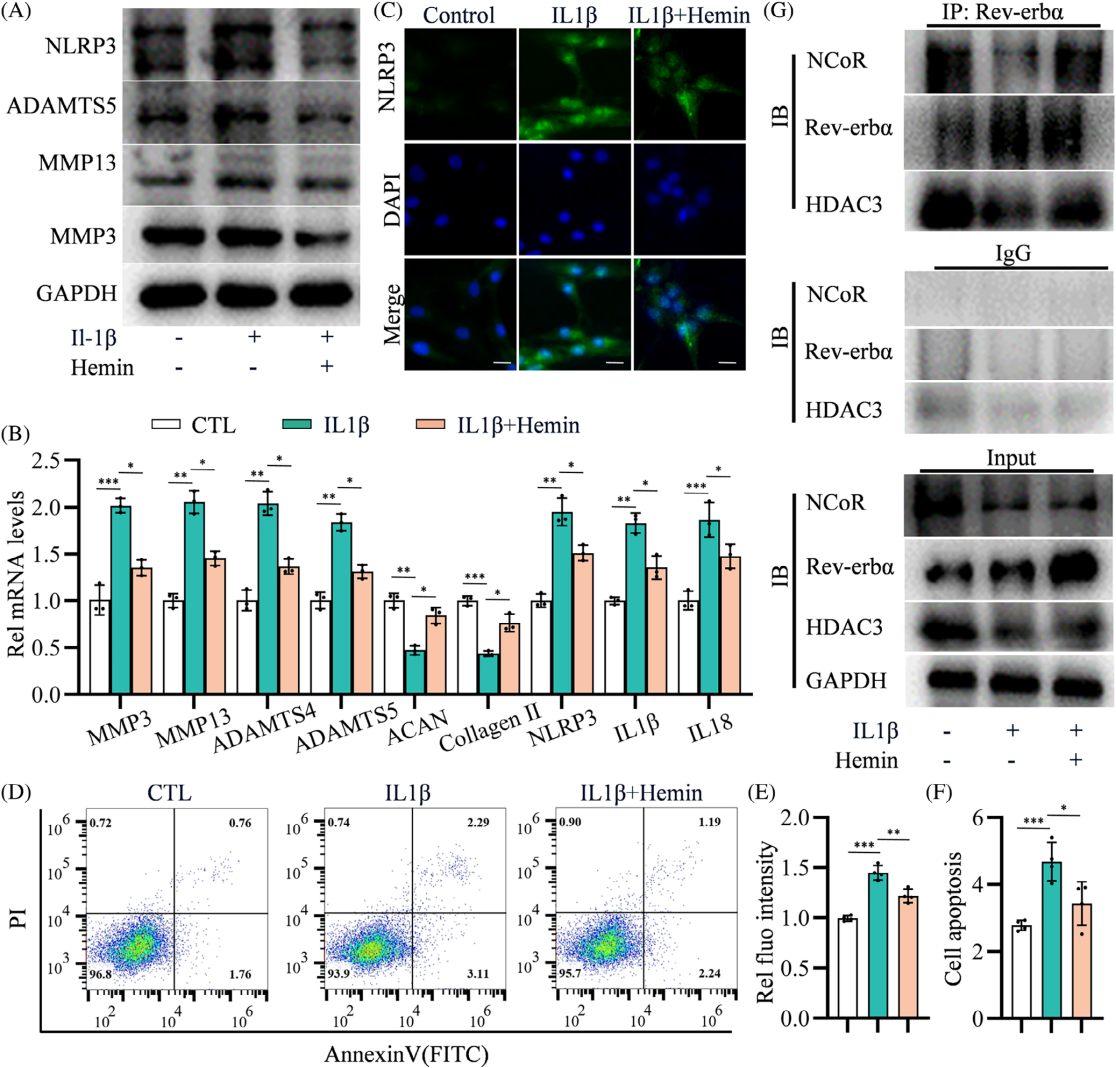
Rev‐erbα recruits the co‐repressors nuclear receptor co‐repressor (NCoR) and histone deacetylase 3 (HDAC3) by hemin to inhibit NLRP3 transcription. (A) Western blot analysis of NLRP3 in NP cells pre‐treated with or without hemin for 1 h and with or without 50 ng/mL rhIL1β for 32 h; *n* = 3. (B) mRNA expression of MMP3, MMP13, ADAMTS4, ADAMTS5, ACAN, collagen II, NLRP3, IL1β, and IL18 in NP cells as treated in (A); *n* = 3. (C,D) Immunofluorescence staining of NLRP3 localization and intensity levels in nucleus pulposus (NP) cells as treated in (A); *n* = 4. Scale bar: 20 μm. (E,F) Apoptosis rate of NP cells assessed using flow cytometry as described in (A); *n* = 4. (G) Co‐immunoprecipitation (CoIP) of hemin to promote Rev‐erbα recruitment of the NCoR and HDAC3 complex. Multivariate analysis of variable was performed to assess statistical significance. *p < 0.05, **p < 0.01, ***p < 0.001.

As the ligand of Rev‐erbα, heme regulates the expression of Rev‐erbα target genes by recruiting the NCoR–HDAC3 co‐repressor complex.^20^, ^21^, ^22^ We conducted immunoprecipitation (IP) with a Rev‐erbα antibody in human NP cells; WB analyses confirmed the interaction between Rev‐erbα and NCoR–HDAC3 (Figure S6A). In this context, we assessed whether exogenous hemin promoted Rev‐erbα repression of the NLRP3 inflammasome in the inflammatory cytokine‐treated NP cells. WB analyses revealed that exogenous hemin significantly reduced the abundances of MMP3, MMP13, ADAMTS5, and NLRP3 in human NP cells cultured with rhIL1β (Figure 5A). qPCR further confirmed the downregulation of catabolism‐ (MMP3, MMP13, ADAMTS4, ADAMTS5) and inflammation‐related genes (NLRP3, IL1β, IL18) and upregulation of metabolism‐related genes (collagen II and ACAN) (Figure 5B). IF staining showed that hemin suppressed NLRP3 expression in rhIL1β‐induced NP cells (Figure 5C,D). Furthermore, FCM analyses revealed that exogenous hemin reduced NP cell apoptosis induced by rhIL1β (Figure 5E,F).

WB analyses revealed that the Rev‐erbα level was decreased in NP cells following a continuous culture with rhIL1β for 14 days (Figure S6B). However, western blotting and qPCR analyses revealed that the Rev‐erbα level was increased in human NP cells treated with specific concentrations of rhIL1β for 2 days (Figures 5G and S6C,D); ECM catabolism markers MMP3 and MMP13 levels were increased under the same culture conditions for 2 days (Figure S6C). Notably, the expression of mRNA encoding d‐aminolevulinate synthase 1 (ALAS1) (marker of heme depletion) was significantly increased in NP cells in the presence of 50 ng/mL rhIL1β for 2 days compared with that in those not exposed to rhIL1β (Figure S6E). CoIP analyses further revealed that Rev‐erbα interacted less with the NCoR–HDAC3 complex in NP cells exposed to rhIL1β for 2 days; however, exogenous hemin promoted the binding of Rev‐erbα to the NCoR–HDAC3 complex (Figure 5G). It was worth emphasizing that BMAL1 abundance was also increased in human NP cells following exposure to certain concentrations of rhIL1β for 2 days (Figure S6F). Taken together, Rev‐erbα inhibits NLRP3 transcription to alleviate IVD degeneration by binding to its ligand heme and recruiting the NCoR–HDAC3 co‐repressor complex.

### 3.7 Intraperitoneal injection of agonist SR9009 attenuates CINS‐induced IVD degeneration

**FIGURE 6:**
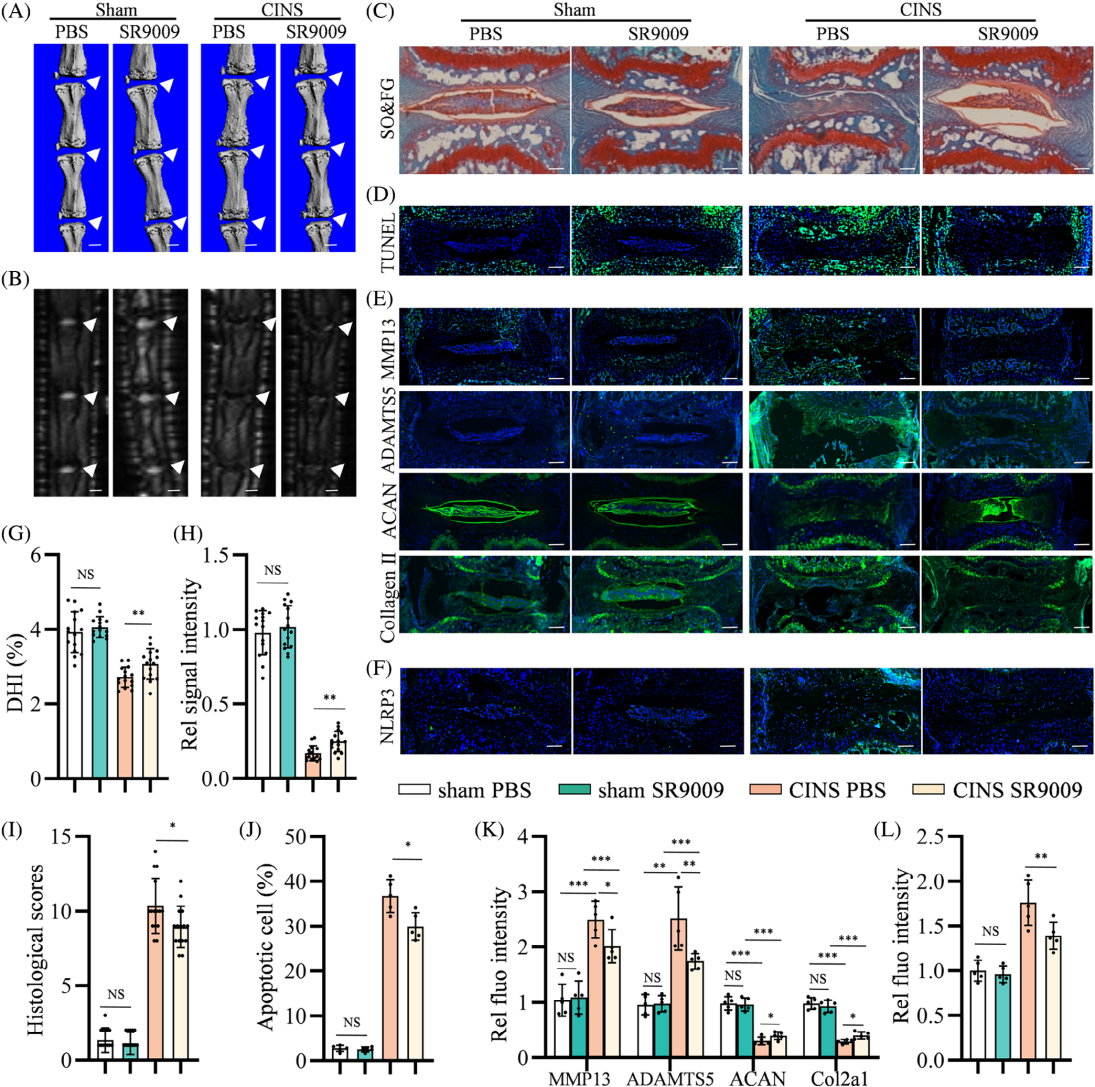
Intraperitoneal injection of agonist SR9009 attenuates coccygeal intervertebral disc (IVD) needle stable (CINS)‐induced IVD degeneration. (A,G) Representative micro‐computed tomography (CT) scans and DHI% of coccygeal IVDs with or without CINS‐induced IVD degeneration treated with SR9009 or polybutylene succinate (PBS); *n* = 5. White triangles: Coccygeal IVDs. Scale bar: 1 mm. (B,H) Representative magnetic resonance imaging (MRI) scan and quantification of the relative signal intensity of IVDs as treated in (A); *n* = 5. White triangles: Coccygeal IVDs. Scale bar: 1 mm. (C,I) Safranin O & Fast Green (SO&FG) staining and histological scores of IVDs as treated in (A); *n* = 5. Scale bar: 100 μm. (D,J) Apoptosis detected using terminal deoxynucleotidyl transferase dUTP nick end labelling (TUNEL) staining of IVDs as treated in (A); *n* = 5. Scale bar: 100 μm. (E,K) Expression of MMP13, ADAMTS5, ACAN, and collagen II detected using immunofluorescence of IVDs as treated in (A); *n* = 5. Scale bar: 100 μm. (F,L) Expression of NLRP3 detected using immunofluorescence of IVDs as treated in (A); *n* = 5. Scale bar: 100 μm. Multivariate analysis was used for more than one variable to assess statistical significance. NS, no statistical significance. *p < 0.05, **p < 0.01, ***p < 0.001.

To develop Rev‐erbα as a clinical therapeutic target, we further investigated the effect elicited by pharmacological activation of Rev‐erbα with SR9009 (a Rev‐erbα agonist) in CINS‐induced mice. Three‐month‐old WT mice subjected to CINS or sham surgery were intraperitoneally injected with SR9009 or PBS; micro‐CT and micro‐MRI showed that CINS‐induced mice that were intraperitoneally injected with SR9009 for 6 weeks exhibited reduced loss of DHI and water contents compared with those injected with PBS (Figure 6A,B,G,H). In addition, SO&FG staining revealed that intraperitoneal administration of SR9009 partly rescued the PG abundance in CINS‐induced IVDs (Figure 6C,I). TUNEL staining showed reduced NP cell apoptosis in CINS‐treated IVDs following intraperitoneal SR9009 administration (Figure 6D,J), and IF staining revealed an increased metabolism and decreased catabolism phenotype characterized with reduced NLRP3 (Figure 6E,F,K,L). However, for IVDs that underwent sham surgery, injection of SR9009 had no statistical differences in ECM homeostasis or cell apoptosis compared with injection of PBS (Figure 6A–J).

We further investigated the therapeutic activation of Rev‐erbα in vitro. WB analyses revealed that SR9009 and GSK4112 (an agonist of Rev‐erbα) partly reduced the expression of MMP3, MMP13, and ADAMTS5 in NP cells induced by rhIL1β (Figure S7A). Additionally, qPCR revealed that SR9009 and GSK4112 downregulated the expression of metabolism markers in NP cells induced by rhIL1β (Figure S7B). FCM analyses showed decreased NP cell apoptosis induced by SR9009 and GSK4112 (Figure S7C,D). The decreased NLRP3 abundance detected using WB analyses and reduced NLRP3, IL1β, and IL18 expression revealed using qPCR indicated that SR9009 and GSK4112 exerted positive effects on NP cells cultured with rhIL1β by inhibiting NLRP3 (Figure S7A–D). Together, these data demonstrate the therapeutic efficacy of Rev‐erbα agonists against rhIL1β‐induced NP cells and CINS‐induced IVD degeneration.

## 4 DISCUSSION

**FIGURE 7:**
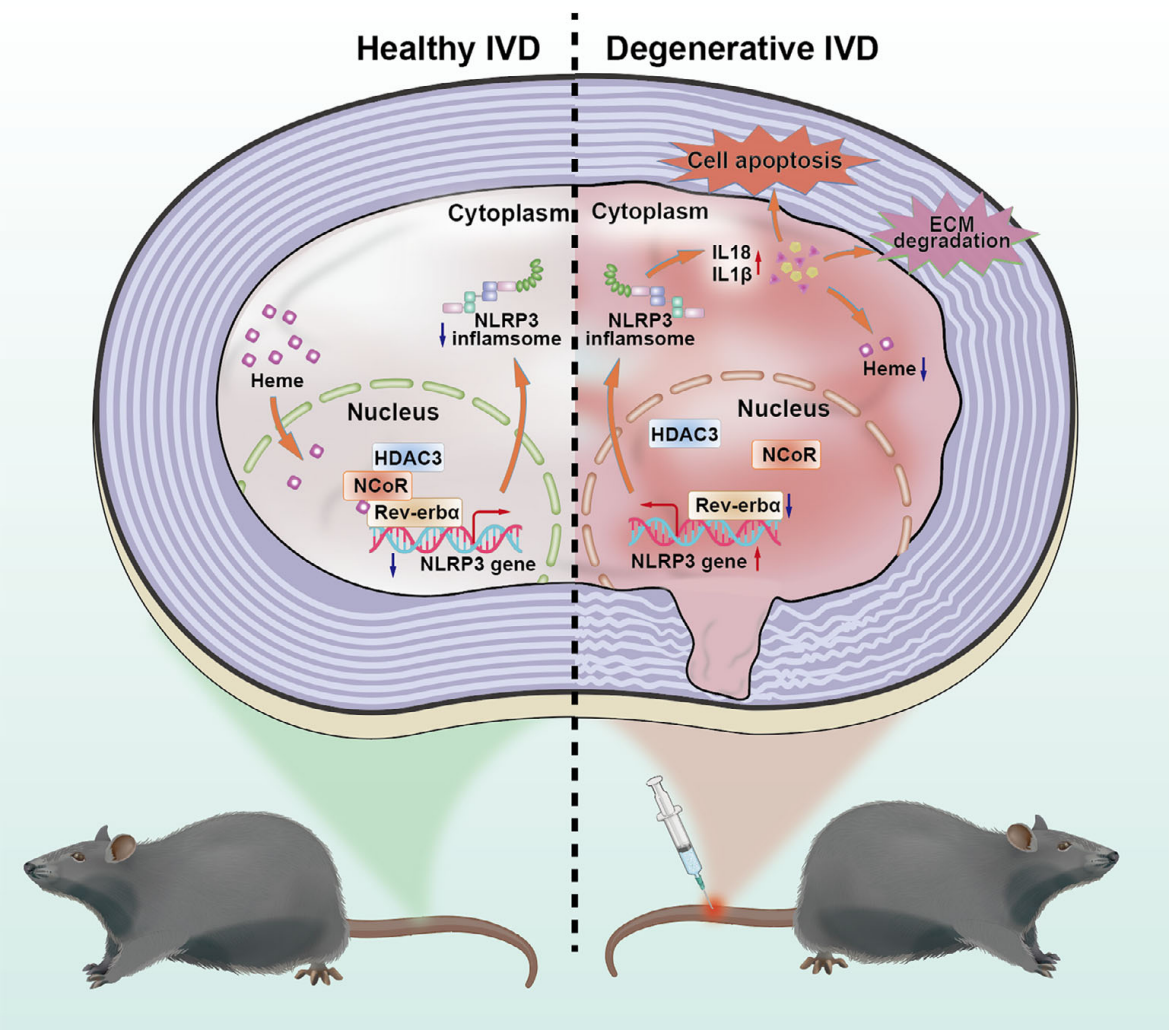
Rev‐erbα deficiency aggravates nucleus pulposus (NP) cell apoptosis and imbalance of extracellular matrix. Specifically, Rev‐erbα alleviates intervertebral disc (IVD) degeneration via its ligand heme binding to nuclear receptor co‐repressor (NCoR) and histone deacetylase 3 (HDAC3) corepressor complex to repress NLRP3 inflammasome activation. Pharmacologic Rev‐erbα activation reduces NP cell apoptosis and improves extracellular matrix (ECM), thereby alleviating IVD degeneration.

The circadian rhythm controls key aspects of IVD physiology and pathophysiology.^1^, ^7^, ^8^, ^15^ The disruption to the circadian clock by aging and inflammation causes IVD degeneration.^1^, ^14^, ^15^ Our study innovatively showed that the core clock component Rev‐erbα was essential for IVD homeostasis, and its deficiency aggravated the progression of IVD degeneration induced by aging and needle puncture. We further demonstrated that Rev‐erbα alleviates IVD degeneration by inhibiting the NLRP3 inflammasome. This process relies on NCoR–HDAC3 complex recruitment by heme. However, the administration of a pharmacological Rev‐erbα agonist relieves CINS‐induced IVD degeneration by inhibiting the NLRP3 inflammasome. Hence, this study provides insights regarding regulating Rev‐erbα in needle puncture and aging‐induced IVD degeneration (Figure 7).

Our study confirmed that Rev‐erbα deficiency aggravated IVD degeneration, and pharmacological activation of Rev‐erbα alleviated IVD degeneration. IVD is a highly rhythmic tissue experiencing daily low‐load recovery cycles that maintain its physiology and homeostasis.^1^ Aging and inflammation disrupt the circadian rhythm of IVDs, causing IVD degeneration. Characterized by disordered BMAL1 expression, misalignment of the circadian rhythm in IVDs is thought to contribute to IVD degeneration and low back pain.^1^, ^7^, ^14^, ^27^ As the core component of the circadian clock, Rev‐erbα regulates key aspects of physiology and pathophysiology, particularly inflammation and immunity.^9^, ^10^, ^16^, ^17^, ^18^ Indeed, Rev‐erbα has been identified as a target for inflammatory diseases, such as osteoarthritis, colitis, hepatitis, neuroinflammation, and pulmonary inflammation.^9^, ^10^, ^11^, ^16^, ^28^, ^29^ This is consistent with the results of this study, showing that Rev‐erbα alleviates IVD degeneration through anti‐inflammatory effects.

Circadian rhythm is associated with aging and longevity, with BMAL1 reportedly moonlighting as a gatekeeper of cellular senescence in primates.^30^, ^31^ A previous study reported that 6‐month‐old mice with an osteoblast‐specific BMAL1 deletion developed spinal kyphoscoliosis deformities.^32^ Meanwhile, in this study, 12‐month‐old Rev‐erbα^−/−^ mice exhibited the aging phenotype with bradykinesia and a large thoracolumbar kyphosis. These results demonstrate the potential of Rev‐erbα in regulating aging and thoracolumbar kyphosis. With the global aging population, adult degenerative scoliosis is increasingly common, proving detrimental to human health.^33^, ^34^, ^35^ Progressive degenerative scoliosis causes severe deformity and excruciating pain, leading to labour loss.^33^, ^34^, ^35^ The aetiology of adult degenerative scoliosis is manifold^34^, ^36^; however, disc degeneration and decreased muscle function are critical causes, demonstrated in our study involving 12‐month‐old Rev‐erbα^−/−^ mice. Rev‐erbα downregulation in muscles reportedly decreases mitochondrial content and oxidative function and upregulates autophagy, consequently aggravating the impaired mitochondrial biogenesis and clearance of disordered organelles, ultimately resulting in compromised exercise capacity.^37^ Furthermore, Rev‐erbα improves skeletal muscle sarcoplasmic reticulum calcium homeostasis by directly binding the sarco/endoplasmic reticulum calcium ATPase promoter and repressing its inhibitor myoregulin.^38^ Meanwhile, pharmacological activation of Rev‐erbα by synthetic ligands revealed its therapeutic effect for improving calcium homeostasis in cells obtained from patients with Duchenne muscular dystrophy and for mitigating the myopathic mouse phenotype. Although the mechanism associated with Rev‐erbα^−/−^ mice having a large thoracolumbar kyphosis remains unclear, our findings provided a new perspective on adult degenerative scoliosis.

Our study further demonstrated that Rev‐erbα alleviated progressive IVD degeneration via repression of the NLRP3 inflammasome. Furthermore, pharmacological inhibition by MCC950 partly rescued Rev‐erbα downregulation‐induced cell apoptosis and disordered metabolism. Increasing evidence suggests that targeting the NLRP3 inflammasome is a potentially effective strategy for treating IVD degeneration.^24^, ^39^ Inflammatory factors, reactive oxygen species, and advanced glycation end products facilitate activation of the NLRP3 inflammasome.^24^, ^39^, ^40^ Meanwhile, certain inhibitors, exosomes, and siRNAs inhibit NLRP3 inflammasome activation and alleviate IVD degeneration.^24^, ^39^, ^41^ A study demonstrated that Rev‐erbα directly repressed the NLRP3 inflammasome in mouse bone marrow‐derived macrophages and regulated IL1β and IL18 maturation and secretion.^9^ In addition, Rev‐erbα directly binds the NLRP3 promoter and inhibits NLRP3 transcription in HEK293T cells, Raw264.7 cells, and mouse primary peritoneal macrophages.^10^ Notably, Rev‐erbα activation indirectly restricts NLRP3 via NF‐κB signalling; these findings are consistent with those of this study, showing that Rev‐erbα targets NLRP3 in NP cells. However, this study is the first to identify NLRP3 as a clock‐targeting gene in human primary NP cells of IVDs. This finding suggests that the rhythmicity of NLRP3 is crucial for its pharmacological action; its rhythmicity as a clock‐controlled gene has been reported in macrophage‐related diseases such as colitis and hepatitis.^9^, ^10^

Rev‐erbα can directly repress transcription of target genes by binding a single ROR response element (RORE) (A/T rich 5′ followed by (A/G)GGTCA half‐site motif).^16^, ^20^, ^22^ Otherwise, Rev‐erbα homodimers bind to two adjacent ROREs or direct 2 bp RORE intervals and recruit the NCoR–HDAC3 co‐repressor complex to repress transcription.^16^, ^17^, ^20^, ^22^, ^42^ We confirmed that Rev‐erbα directly binds the NCoR–HDAC3 co‐repressor complex to suppress NLRP3 activation in human primary NP cells. Notably, Rev‐erbα expression was decreased in NP cells following exposure to rhIL1β for 14 days; however, its expression increased following rhIL1β culture for 2 days. We speculate that the increased Rev‐erbα expression failed to inhibit NLRP3 transcription owing to heme depletion. As was demonstrated by increased ALSA1 expression in the NP cells cultured with rhIL1β for 2 days, temporarily increased Rev‐erbα in NP cells failed to bind to the NCoR–HDAC3 co‐repressor complex to repress activation of the NLRP3 inflammation. As the rate‐limiting enzyme for the heme metabolism, the expression of heme oxygenase 1 reportedly increases and then decreases in NP cells treated with H_2_O_2_.^43^ These results are similar to ours following heme depletion in NP cells cultured for a short period with rhIL1β.

We also observed increased BMAL1 expression in the NP cells cultured with rhIL1β for 2 days. Rev‐erbα represses BMAL1 to generate circadian oscillation in the transcriptional–translational feedback loop system.^13^, ^29^, ^30^ Therefore, failure to inhibit BMAL1 transcription indicated that the increased Rev‐erbα in NP cells exposed to rhIL1β for 2 days was inactive. Similarly, a previous study demonstrated that heme depletion significantly increases BMAL1 expression owing to Rev‐erbα suppression.^20^, ^42^ Accordingly, we hypothesize that the temporary increase in Rev‐erbα serves as physiological feedback to recruit the NCoR–HDAC3 co‐repressor complex and maintain homeostasis. However, heme depletion prevented Rev‐erbα from combining with the NCoR–HDAC3 co‐repressor complex. Meanwhile, exogenous hemin alleviated the degenerative IVD phenotype by promoting metabolism, suppressing catabolism, and reducing cell apoptosis. More studies are necessary to investigate the dynamic process of Rev‐erbα in response to stimuli.

In conclusion, Rev‐erbα maintains IVD homeostasis and protects IVDs against degeneration by suppressing NLRP3 inflammasome activation. This physiological effect depends on its binding to heme and subsequent recruitment of the NCoR–HDAC3 co‐repressor complex. Activation of Rev‐erbα may become a valuable clinical therapeutic strategy to treat IVD degeneration and its related diseases.

## AUTHOR CONTRIBUTIONS

Q.Z. performed the most experiments, analysed data, and drafted the article. X.P. performed the experiments and analysed data. Z.Q. performed the most experiments and revised the article. H.C. participated in the experiments and collected the samples. N.W. participated in the experiments and analysed data. S.W. participated in the experiments and collected the samples. H.Z. participated in the experiments. Z.F. revised the article and supervised the study. Z.Z. revised the article and supervised the study. B.W. collected and processed the samples. Y.Q. designed and supervised the study. X.S. designed and supervised the study.

## FUNDING INFORMATION

This work was supported by the Jiangsu Provincial Medical Innovation Center of Orthopaedic Surgery (Grant No. CXZX202214), and Postgraduate Research & Practice Innovation Program of Jiangsu Province (Grant No. KYCX22_3709).

## CONFLICT OF INTEREST STATEMENT

The authors declare no competing interests.

## PATIENT CONSENT STATEMENT

The enrolled patients signed informed consent before surgery.

## Supporting information


**Video S1a.** Motion video of 12‐month‐old WT mice.

**Video S1b.** Motion video of 12‐month‐old Rev‐erbα−/− mice.

**Data S1.** Supporting information.

## Data Availability

The presented dataset is available by contacting the corresponding author.
