# Supplementary material for: Nuclear receptor Rev‐erbα alleviates intervertebral disc degeneration by recruiting NCoR–HDAC3 co‐repressor and inhibiting NLRP3 inflammasome
Source: Cell Prolif. 2024 Jul 24;57(12):e13720. doi: 10.1111/cpr.13720 (PMC11628727; doi:10.1111/cpr.13720)
Supplement: Supplementary file 3 — Data S1. Supporting information. [file CPR-57-e13720-s003.doc]

**Nuclear receptor Rev-erbα alleviates intervertebral disc degeneration via recruiting NCoR-HDAC3 co-repressor and inhibiting NLRP3 inflammasome**

**AUTHORS:** Qingshuang Zhou, MD^1^, Xiaojiang Pu, MD^2^, Zhuang Qian, MD^1,2^, Haojie Chen, MM^2^, Nannan Wang, MM^2^, Sinian Wang, MD^2^, Haicheng Zhou, MM^1,2^, Zhenhua Feng, MD^1,2^, Zezhang Zhu, MD^1,2^, Bin Wang, MD^1,2^, Yong Qiu, MD^1,2^*, Xu Sun, MD^1,2^*

^1^Division of Spine Surgery, Department of Orthopedic Surgery, Nanjing Drum Tower Hospital Clinical College of Jiangsu University, Nanjing, China

^2^Division of Spine Surgery, Department of Orthopedic Surgery, Nanjing Drum Tower Hospital, Affiliated Hospital of Medical School, Nanjing University, Nanjing, China

**Supplementary figures and tables**

**
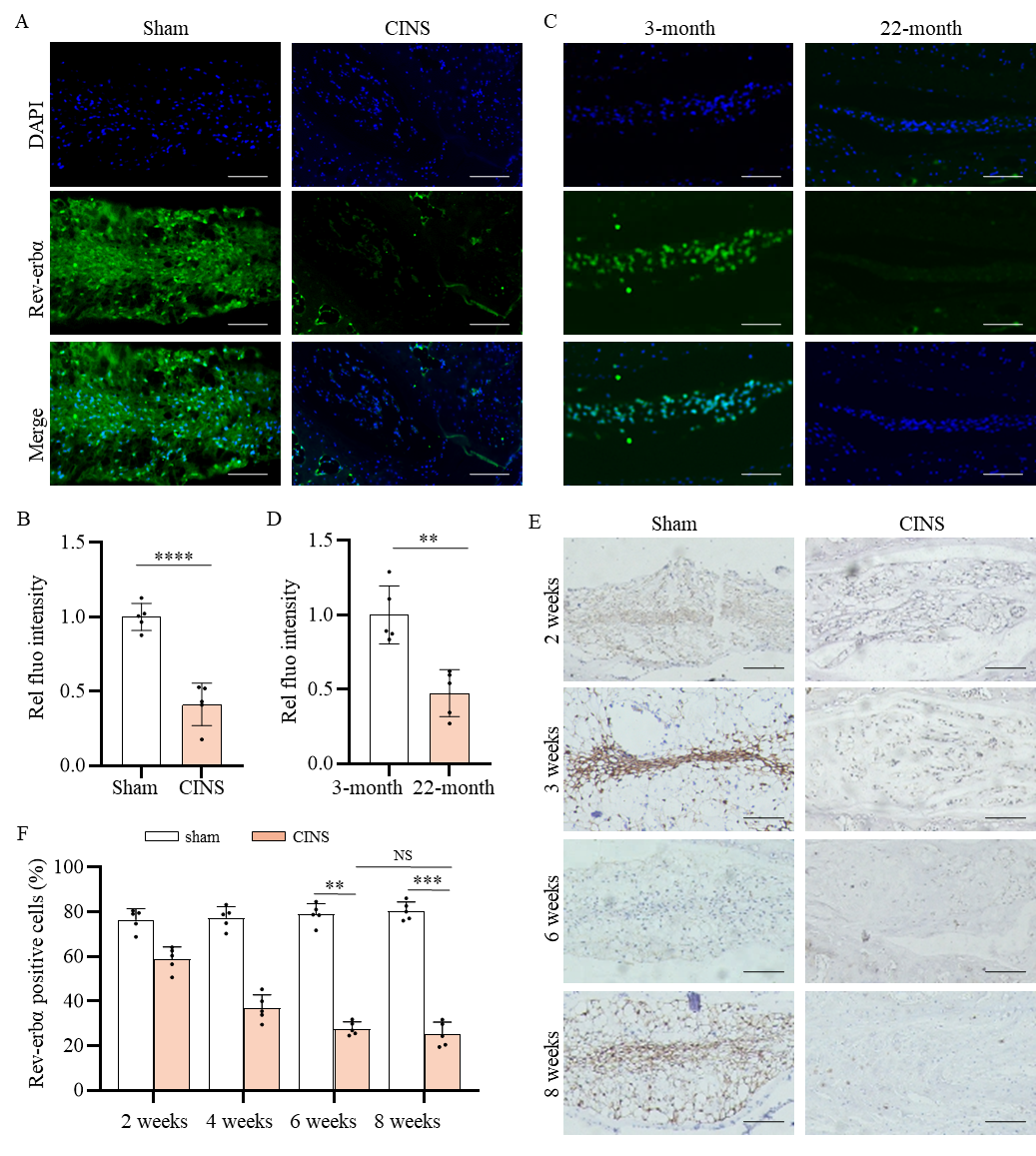
**

**Supplementary Fig. 1** Expression of Rev-erbα assessed using immunostaining staining. **(A, B)** Immunofluorescence (IF) staining of Rev-erbα for sham and coccygeal IVD needle stable (CINS)-induced IVD degradation for 6 weeks; n = 5. Scale bar: 100 μm. **(C, D)** IF staining of Rev-erbα for young (3-month-old) and aged (22-month-old) mouse IVDs; n = 5. Scale bar: 100 μm. **(E, F)** Immunohistochemistry (IHC) staining of Rev-erbα in CINS-induced IVDs after 2, 4, 6, 8 weeks; n = 5. Scale bar: 100 μm. Student’s *t*-test was used to assess statistical significance. NS, no statistical significance. **P < 0.01, ***P < 0.001.

**
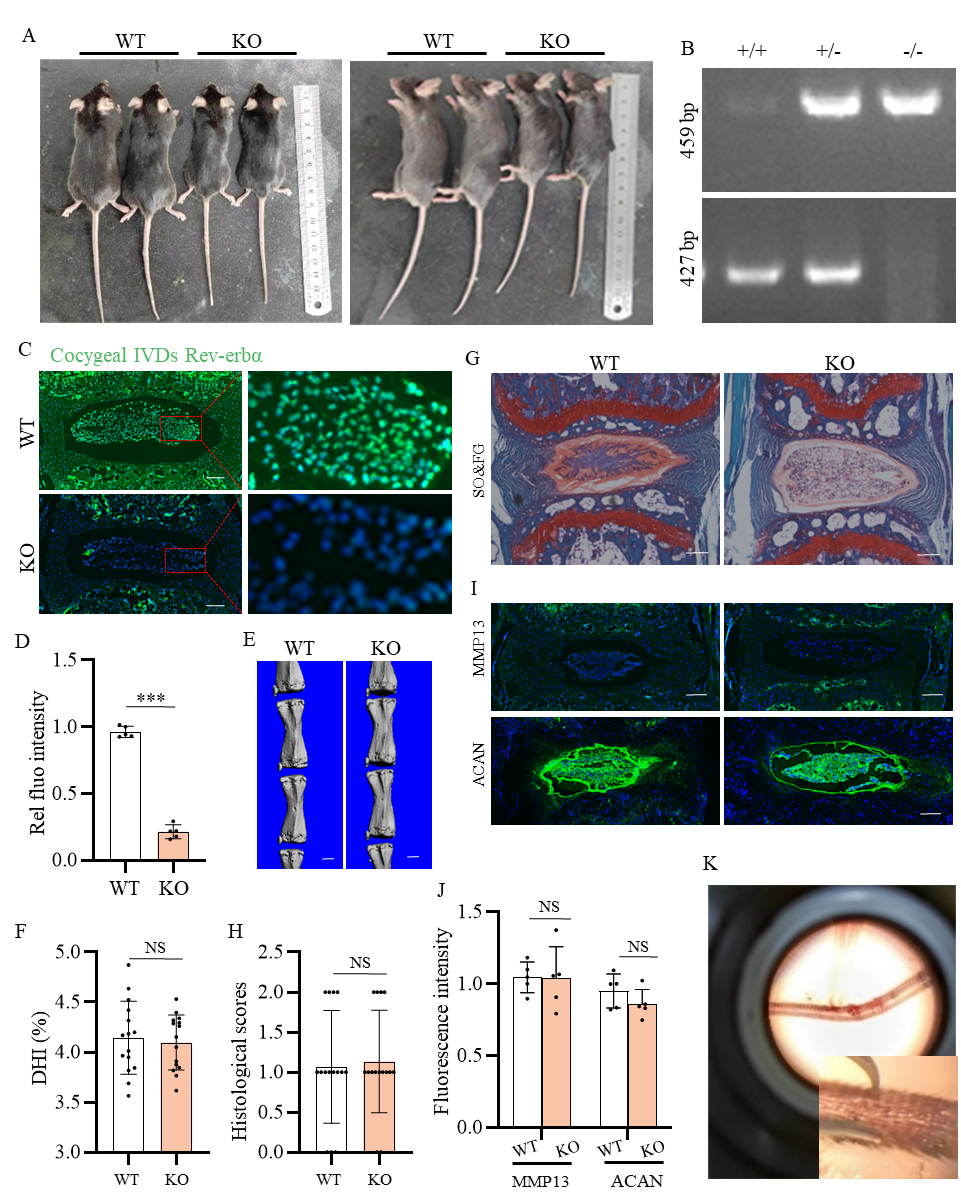
**

**Supplementary Fig. 2** Phenotype of Rev-erbα knockout (KO) mice**.** **(A)** Representative sizes of wildtype (WT) and Rev-erbα^-/-^ mice. **(B)** Genotyping PCR for WT mice (+/+), heterozygotes (+/-), and homozygotes (KO, -/-). **(C, D)** IF staining of Rev-erbα in coccygeal IVDs of 4-month-old WT and Rev-erbα^-/-^ mice; n = 5. Scale bar: 100 μm. **(E, F)** Representative micro-CT scans and disc height index (DHI) of 4-month-old WT and Rev-erbα^-/-^ mice. Scale bar: 1 mm. **(G, H)** SO&FG staining and histological scores of coccygeal IVDs from 4-month-old WT and Rev-erbα^-/-^ mice; n = 5. Scale bar: 100 μm. **(I, J)** IF staining of MMP13 and ACAN for coccygeal IVDs of WT and Rev-erbα^-/-^ mice with or without CINS; n = 5. Scale bar: 100 μm. **(K)** Photos of murine coccygeal IVD needle stable (CINS) surgery. Student’s *t*-test was used to assess statistical significance. NS, no statistical significance. *P < 0.05, **P < 0.01, ***P < 0.001.

**
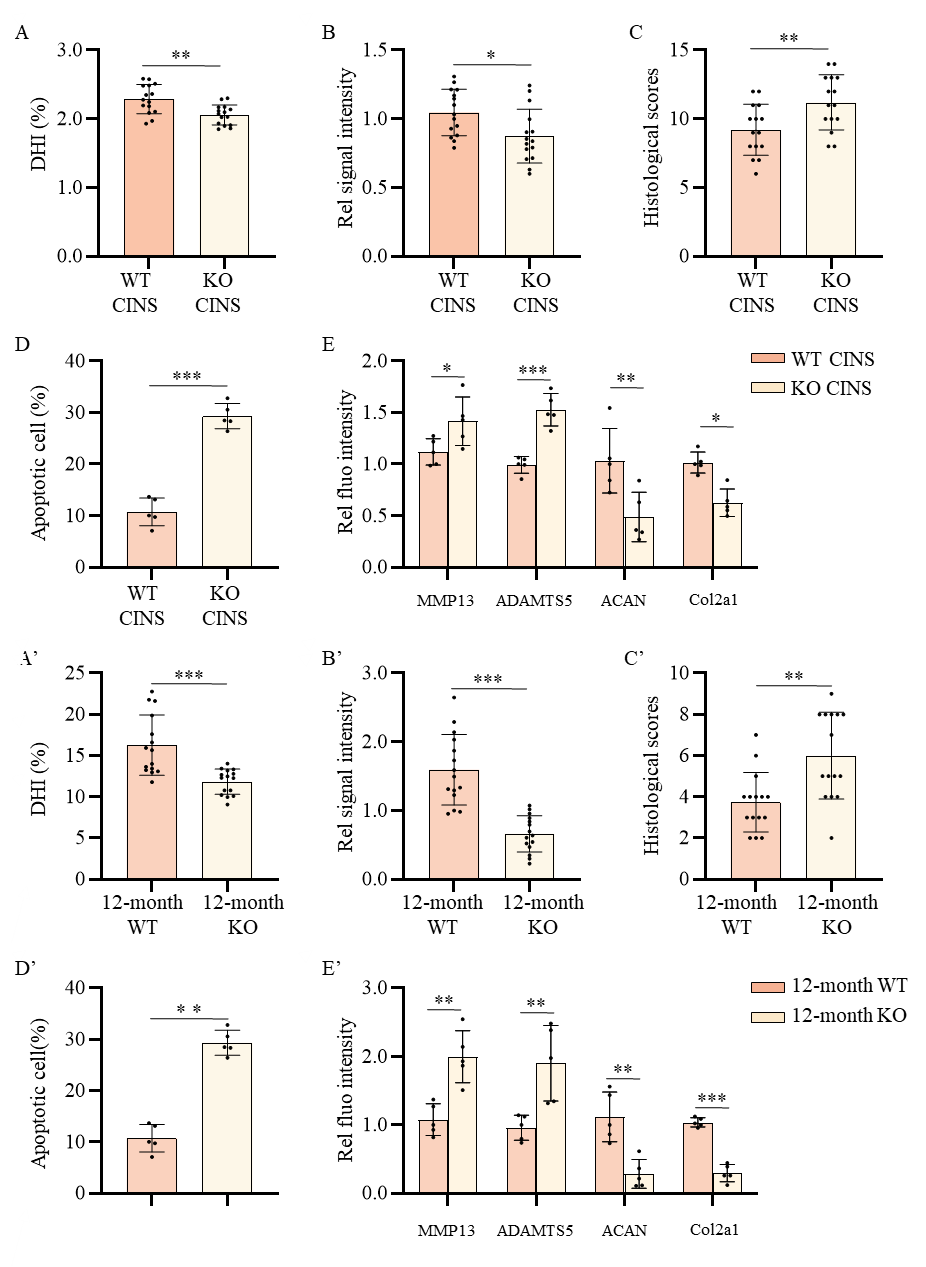
**

**Supplementary Fig. 3** Quantification of detected markers in Fig. 2. **(A)** DHI revealed by micro-CT scan of CINS-induced coccygeal vertebrae from WT and Rev-erbα^-/-^ mice; n = 5. (**B)** Quantification of the relative signal intensity revealed by micro-MRI scan of CINS-induced coccygeal vertebrae from WT and Rev-erbα^-/-^ mice; n = 5. (**C)** Histological scores revealed by SO&FG staining of CINS-induced coccygeal vertebrae from WT and Rev-erbα^-/-^ mice; n = 5. (**D)** Percentage of apoptotic cells revealed by TUNEL staining of CINS-induced coccygeal vertebrae from WT and Rev-erbα^-/-^ mice; n = 5. (**E)** Quantifying IF staining of MMP13, ADAMTS5, ACAN, and Collagen Ⅱ for CINS-induced coccygeal vertebrae of WT and Rev-erbα^-/-^ mice; n = 5. **(A’)** DHI revealed using micro-CT scans of lumbar IVDs (L3/4, L4/5, L5/6) from 12-month-old WT and Rev-erbα^-/-^ mice; n = 5. **(B’)** Quantification of the relative signal intensity revealed using a micro-MRI scan of lumbar IVDs (L3/4, L4/5, L5/6) from 12-month-old WT and Rev-erbα^-/-^ mice; n = 5. **(C’)** Histological scores revealed by SO&FG staining of lumbar IVDs from 12-month-old WT and Rev-erbα^-/-^ mice; n = 5. **(D’)** Percentage of apoptotic cells revealed using TUNEL staining of NP tissues from 12-month-old WT and Rev-erbα^-/-^ mice; n = 5. Scale bar: 100 μm. **(E’)** Quantifying IF staining of MMP13, ADAMTS5, ACAN, and Collagen Ⅱ for 12-month-old WT and Rev-erbα^-/-^ mice; n = 5. Scale bar: 100 μm. Student’s *t*-test was used to assess statistical significance. *P < 0.05, **P < 0.01, ***P < 0.001.


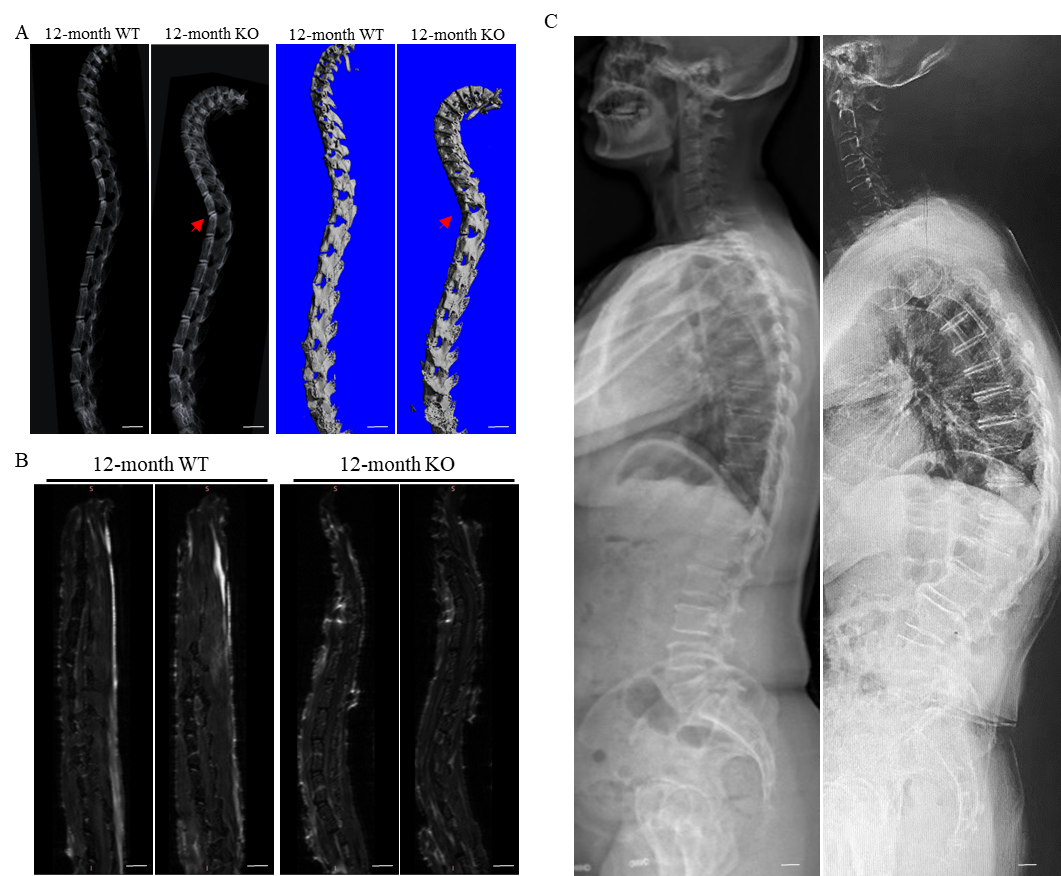


**Supplementary Fig. 4** Rev-erbα deletion results in large thoracolumbar kyphosis. **(A)** Representative micro-CT scans of 12-month-old WT and Rev-erbα^-/-^ mouse lumbar vertebrae. Scale bar: 5 mm. **(B)** Representative micro-MRI scans of WT and 12-month-old mouse lumbar vertebrae. Scale bar: 2 cm. **(C)** Representative X-ray radiographs of patients with or without a large thoracolumbar kyphosis.


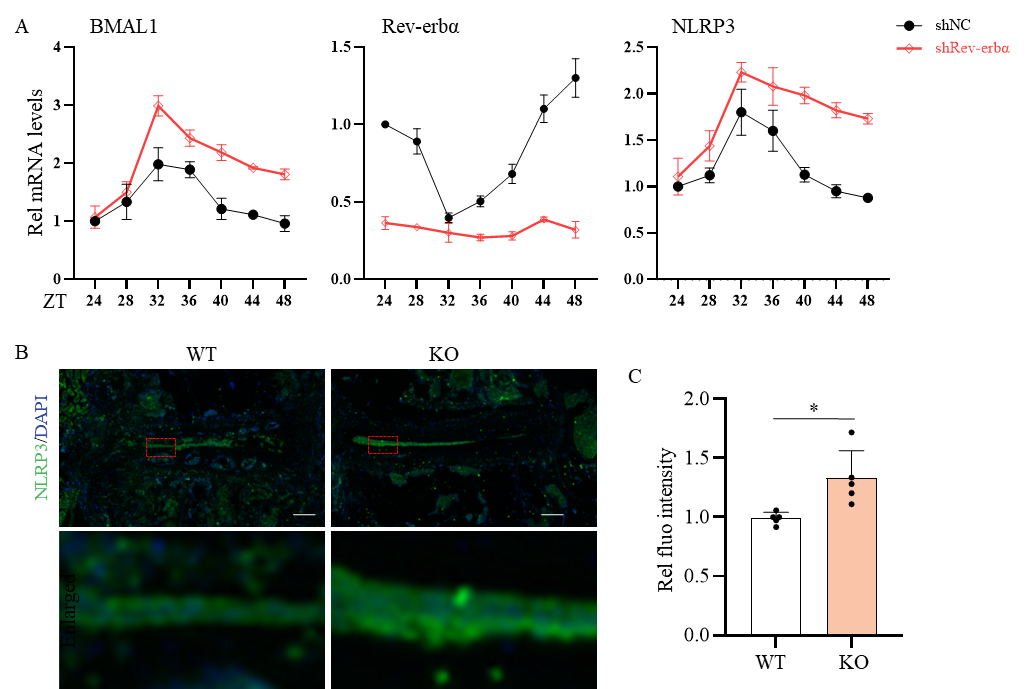


**Supplementary Fig. 5** Identification of *NLRP3* as the Rev-erbα clock-controlled gene in the nucleus pulposus of human IVDs. **(A)** mRNA circadian expression of Rev-erbα, BMAL1, and NLRP3 in human primary NP cells transfected with negative control (shNC) or Rev-erbα knockdown lentivirus (shRev-erbα); n = 3. **(B, C)** NLRP3 expression detected using IF staining of IVDs from WT and Rev-erbα^-/-^ mice; n = 5. Scale bar: 100 μm. Student’s *t*-test was used to assess statistical significance. *P < 0.05.


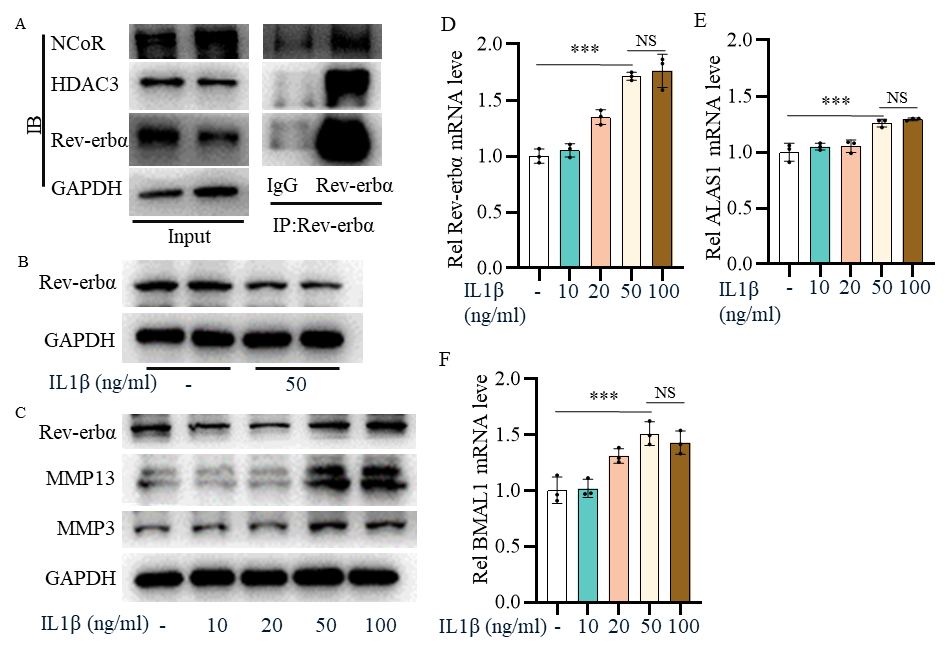


**Supplementary Fig. 6** Rev-erbα functions as a transcription repressor depending on recruitment of the NCoR–HDAC3 complex by heme. **(A)** Immunoprecipitation (IP) of Rev-erbα recruitment of NCoR and HDAC3 complex in human NP cells. **(B)** Western blot analyses of Rev-erbα, MMP3, and MMP13 in human NP cells treated with different rhIL1β concentrations for 2 days. **(C)** Western blot analyses of Rev-erbα in human NP cells treated with 50 ng/mL rhIL1β for 14 days; n = 3. **(D)** qPCR of Rev-erbα in human NP cells as treated in (c); n = 3. **(E)** qPCR of ALAS1 in human NP cells as treated in (c). **(F)** qPCR of BMAL1 in human NP cells as treated in (c); n = 3. Student’s *t*-test was used to assess statistical significance. NS, no statistical significance. ***P < 0.001.


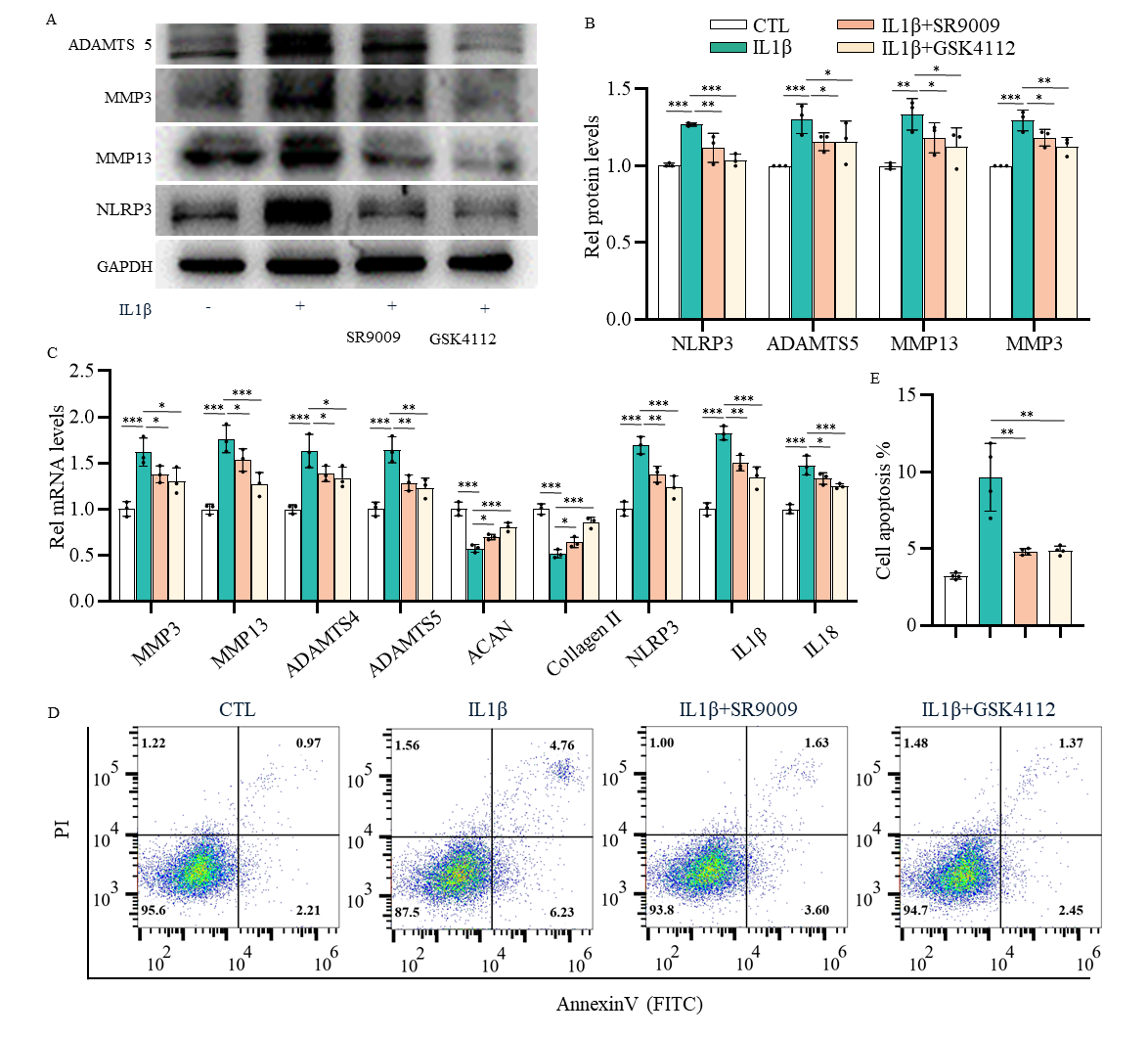


**Supplementary Fig. 7** Rev-erbα activation inhibits ECM catabolism and cell apoptosis in NP cells through the NLRP3 inflammasome. **(A)** Western blot analyses of NLRP3 in NP cells pretreated with or without agonist SR9009 or GSK4112 for 1 h and treated with or without rhIL1β for 32 h; n = 3. **(B)** mRNA expression of *MMP3, MMP13, ADAMTS4, ADAMTS5, ACAN*, Collagen Ⅱ, *NLRP3, IL1*β*,* and *IL18* in NP cells treated as in (a); n = 3. **(C, D)** Flow cytometry analyses of apoptosis rate of NP cells treated as in (a); n = 4. Student’s *t*-test or multivariate ANOVA was performed to assess statistical significance. *P < 0.05, **P < 0.01, ***P < 0.001.

**Supplementary** **Videos**

Motion video of 12-month-old wildtype (WT) and Rev-erbα^-/-^ mice. **(a)** Motion video of 12-month-old WT mice. **(b)** Motion video of 12-month-old Rev-erbα^-/-^ mice.

**Supplementary Table 1 Patient baseline characteristics.**

| N0. | Age (years) | Gender | Diagnosis | Level | Pfirrmann grade |
| --- | --- | --- | --- | --- | --- |
| 1 | 48 | ♀ | lumbar stenosis | L4/5 | II |
| 2 | 39 | ♀ | lumbar disc herniation | L5/S1 | III |
| 3 | 19 | ♂ | lumbar spondylolysis | L5/S1 | II |
| 4 | 18 | ♂ | lumbar stenosis | L4/5 | II |
| 5 | 29 | ♀ | lumbar spondylolysis | L5/S1 | II |
| 6 | 31 | ♀ | lumbar disc herniation | L4/5 | III |
| 7 | 25 | ♀ | lumbar disc herniation | L5/S1 | II |
| 8 | 17 | ♂ | lumbar spondylolysis | L5/S1 | II |
| 9 | 16 | ♂ | hemivertebrae | L4/5 | II |
| 10 | 19 | ♂ | lumbar spondylolysis | L5/S1 | II |
| 11 | 23 | ♀ | lumbar disc herniation | L4/5 | II |
| 12 | 30 | ♀ | lumbar disc herniation | L4/5 | II |
| 13 | 32 | ♀ | lumbar disc herniation | L4/5 | III |
| 14 | 70 | ♂ | lumbar disc herniation | L4/5 | V |
| 15 | 53 | ♂ | lumbar disc herniation | L4/5 | IV |
| 16 | 54 | ♂ | lumbar disc herniation | L5/S1 | IV |
| 17 | 69 | ♀ | lumbar disc herniation | L4/5 | V |
| 18 | 65 | ♀ | lumbar stenosis | L4/5 | IV |
| 19 | 64 | ♂ | lumbar disc herniation | L5/S1 | V |
| 20 | 68 | ♀ | lumbar stenosis | L4/5 | V |
| 21 | 58 | ♀ | lumbar disc herniation | L4/5 | IV |
| 22 | 72 | ♀ | lumbar disc herniation | L4/5 | V |
| 23 | 68 | ♂ | lumbar stenosis | L5/S1 | V |
| 24 | 66 | ♂ | lumbar stenosis | L5/S1 | IV |
| 24 | 66 | ♂ | lumbar stenosis | L5/S1 | IV |
| 25 | 63 | ♀ | lumbar stenosis | L4/5 | V |
| 26 | 57 | ♀ | lumbar stenosis | L4/5 | IV |
| 27 | *56* | ♀ | lumbar stenosis | L5/S1 | V |
| 28 | 61 | ♂ | lumbar stenosis | L4/5 | IV |
| 29 | 42 | ♂ | lumbar stenosis | L5/S1 | II |
| 30 | 42 | ♂ | lumbar disc herniation | L4/5 | III |
| 31 | 21 | ♂ | lumbar disc herniation | L5/S1 | II |
| 32 | 28 | ♂ | lumbar disc herniation | L4/5 | II |
| 33 | 37 | ♀ | lumbar spondylolysis | L5/S1 | II |
| 34 | 58 | ♀ | lumbar stenosis | L3-L5 | III |
| 35 | 15 | ♀ | lumbar spondylolysis | L5/S1 | II |
| 36 | 42 | ♀ | lumbar disc herniation | L4/5 | III |
| 37 | 37 | ♀ | lumbar spondylolysis | L5/S1 | III |
| 38 | 21 | ♀ | hemivertebrae | L5/S1 | I |
| 39 | 15 | ♀ | hemivertebrae | L4/5 | I |
| 40 | 14 | ♂ | lumbar disc herniation | L5/S1 | III |
| 41 | 18 | ♂ | lumbar spondylolysis | L5/S1 | II |

**Supplementary Table 2** Primer sequences for genotyping.

| **Gene** | **Forward (5’-3’)** | **Reverse (5’-3’)** |
| --- | --- | --- |
| Knock out | CTGCAACTCATCTGAGTCTTATCTG | TCACGTTGAACAACGATGCAAA |
| Wildtype allele | CTGCAACTCATCTGAGTCTTATCTG | CATTAAGCTTCTGGTTTGGGTGG |

**Supplementary Table 3** Antibody information.

| Antibody | Company | Catalog # | Application/Dilution |
| --- | --- | --- | --- |
| Rev-erbα | CST | 13418 | IP ( 1:100) |
| Rev-erbα | Santacruz | sc-100910 | WB (1:500)  IF (1:200)  IHC (1:100)  WB (1:200) |
| Rabbit (DA1E) mAb IgG XP | CST | 3900 | Isotype control |
| HDAC3 | Affinity | AF6016 | WB (1:500) |
| NCoR | Santacruz | sc-515934 | WB (1:200) |
| MMP3 | Proteintech | 66338-1-Ig | WB (1:5000) |
| MMP13 | Abcam | ab39012 | WB (1:3000) |
|  |  |  | IF (1:200) |
| ADAMTS5 | Affinity | DF13268 | WB (1:500) |
| ADAMTS5 | Abcam | ab246975 | IF (1:400) |
| Collagen Ⅱ | Abcam | ab34712 | IF (1:100) |
| Aggrecan | Sigma-Aldrich | AB1031 | IF (1:50) |
| NLRP3 | Affinity | DF7438 | WB (1:Supplementary Table 34OVA) tests000000000000000000000000000000000000000000000000000000000000000000000000000000000000000000000000Supplementary Table 34OVA) tests000000000000000000000000000000000000000000000000000000000000000000000000000000000000000000000000500) |
|  |  |  | IF (1:100) |
| GAPDH | Proteintech | GB15004-100 | WB (1:1000) |
| Goat anti-rabbit IgG | Biosharp | BL003A | WB (1:5000) |
| Goat anti-mouse IgG | Biosharp | BL001A | WB (1:5000)  IHC (1:400) |
| Goat anti-rabbit IgG | Invitrogen | A16024 | IF (1:400) |
| Goat anti-mouse IgG | Invitrogen | A-11001 | IF (1:400) |

**Supplementary Table 4 The sequences of primers used in this study.**

| Genes | Primer sequence (forward primers) | Primer sequence (reverse primers) |
| --- | --- | --- |
| GAPDH | GGAGCGAGATCCCTCCAAAAT | GGCTGTTGTCATACTTCTCATGG |
| NCoR | ACACCGCAGTATTGTCCAAAT | CACCTGGTTTGTCTTGATGTTCT |
| HDAC3 | CCTGGCATTGACCCATAGCC | CTCTTGGTGAAGCCTTGCATA |
| BMAL1 | AAGGGAAGCTCACAGTCAGAT | GGACATTGCGTTGCATGTTGG |
| Rev-erbα | TGGACTCCAACAACAACACAG | GATGGTGGGAAGTAGGTGGG |
| MMP3 | AGTCTTCCAATCCTACTGTTGCT | TCCCCGTCACCTCCAATCC |
| MMP13 | ACTGAGAGGCTCCGAGAAATG | GAACCCCGCATCTTGGCTT |
| ADAMTS4 | GAGGAGGAGATCGTGTTTCCA | CCAGCTCTAGTAGCAGCGTC |
| ADAMTS5 | GAACATCGACCAACTCTACTCCG | CAATGCCCACCGAACCATCT |
| Collagen 2 | TGGACGATCAGGCGAAACC | GCTGCGGATGCTCTCAATCT |
| Aggrecan | ACTCTGGGTTTTCGTGACTCT | ACACTCAGCGAGTTGTCATGG |
| NLRP3 | GATCTTCGCTGCGATCAACAG | CGTGCATTATCTGAACCCCAC |
| IL-1β | ATGATGGCTTATTACAGTGGCAA | GTCGGAGATTCGTAGCTGGA |
| IL-18 | TCTTCATTGACCAAGGAAATCGG | TCCGGGGTGCATTATCTCTAC |
| ALAS1 | CGCCGCTGCCCATTCTTAT | TCTGTTGGACCTTGGCCTTAG |
